# Supplementary material for: Longitudinal association between food frequency and changes in body mass index: a prospective cohort study
Source: BMJ Open. 2020 Sep 3;10(9):e037057. doi: 10.1136/bmjopen-2020-037057 (PMC7473622; doi:10.1136/bmjopen-2020-037057)
Supplement: Author's manuscript [file bmjopen-2020-037057.draft_revisions.pdf]

BMJ Open

BMJ Open is committed to open peer review. As part of this commitment we make the peer review history of every article we publish publicly available.

When an article is published we post the peer reviewers' comments and the authors' responses online. We also post the versions of the paper that were used during peer review. These are the versions that the peer review comments apply to.

The versions of the paper that follow are the versions that were submitted during the peer review process. They are not the versions of record or the final published versions. They should not be cited or distributed as the published version of this manuscript.

BMJ Open is an open access journal and the full, final, typeset and author-corrected version of record of the manuscript is available on our site with no access controls, subscription charges or pay-per-view fees (http://bmjopen.bmj.com).

If you have any questions on BMJ Open's open peer review process please email info.bmjopen@bmj.com

BMJ Open

Meal frequency and changes in body mass index: findings from the Young Lives Study in Peru

Journal:	*BMJ Open*
Manuscript ID	bmjopen-2020-037057
Article Type:	Original research
Date Submitted by the Author:	17-Jan-2020
Complete List of Authors:	Bernabe-Ortiz, Antonio; Universidad Peruana Cayetano Heredia, CRONICAS Centre of Excellence in Chronic Diseases<br>Carrillo-Larco, Rodrigo; Universidad Peruana Cayetano Heredia, CRONICAS Center of Excellence; CRONICAS Center of Excellence in Chronic Diseases
Keywords:	EPIDEMIOLOGY, NUTRITION & DIETETICS, PUBLIC HEALTH

SCHOLARONE™
Manuscripts

I, the Submitting Author has the right to grant and does grant on behalf of all authors of the Work (as defined in the below author licence), an exclusive licence and/or a non-exclusive licence for contributions from authors who are: i) UK Crown employees; ii) where BMJ has agreed a CC-BY licence shall apply, and/or iii) in accordance with the terms applicable for US Federal Government officers or employees acting as part of their official duties; on a worldwide, perpetual, irrevocable, royalty-free basis to BMJ Publishing Group Ltd ("BMJ") its licensees and where the relevant Journal is co-owned by BMJ to the co-owners of the Journal, to publish the Work in this journal and any other BMJ products and to exploit all rights, as set out in our licence.

The Submitting Author accepts and understands that any supply made under these terms is made by BMJ to the Submitting Author unless you are acting as an employee on behalf of your employer or a postgraduate student of an affiliated institution which is paying any applicable article publishing charge ("APC") for Open Access articles. Where the Submitting Author wishes to make the Work available on an Open Access basis (and intends to pay the relevant APC), the terms of reuse of such Open Access shall be governed by a Creative Commons licence – details of these licences and which Creative Commons licence will apply to this Work are set out in our licence referred to above.

Other than as permitted in any relevant BMJ Author's Self Archiving Policies, I confirm this Work has not been accepted for publication elsewhere, is not being considered for publication elsewhere and does not duplicate material already published. I confirm all authors consent to publication of this Work and authorise the granting of this licence.

For peer review only

Meal frequency and changes in body mass index: findings from the Young Lives Study in Peru

Antonio Bernabe-Ortiz, MD, MPH, PhD[1,2]; Rodrigo M. Carrillo-Larco, MD[1,3,4]

1. CRONICAS Center of Excellence in Chronic Diseases, Universidad Peruana Cayetano Heredia, Lima, Peru
2. Universidad Cientifica del Sur, Lima, Perú
3. Department of Epidemiology and Biostatistics, School of Public Health, Imperial College London, London, UK
4. Centro de Estudios de Población, Universidad Católica los Ángeles de Chimbote (ULADECH-Católica), Chimbote, Peru

For peer review only

Short title:

Meal frequency and body mass index

E-mails:

Antonio Bernabe-Ortiz: Antonio.Bernabe@upch.pe

Rodrigo M. Carrillo-Larco: Rodrigo.Carrillo@upch.pe

Corresponding autor:

Antonio Bernabe-Ortiz, MD, MPH, PhD

CRONICAS Center of Excellence in Chronic Diseases, Universidad Peruana Cayetano Heredia

Av. Armendariz 445, Miraflores, Lima, Peru

E-mail: Antonio.Bernabe@upch.pe

Phone: 51-1-241-6978

Word count:

Abstract: 227 words

Manuscript (from Introduction to References): 3000 words (total: 4318 words)

For peer review only

ABSTRACT

Objectives: Analyzing data of the Young Lives Study in Peru, we aimed at assessing the association between daily meal frequency and BMI changes between 2006 and 2016.

Design: Secondary analysis of a prospective ongoing cohort study.

Setting: 20 sentinel sites in Peru

Participants: Children enrolled in the younger cohort of the Young Lives Study. We used information from the second (2006-07), third (2009-10), fourth (2013-14), and fifth (2016-17) rounds of the younger cohort in Peru.

Primary and secondary outcomes: Body mass index (BMI) as well as BMI-for-age z-score, both as numerical variables.

Results: Data from 1948 children, mean age 4.3 (SD: 0.3) years and 966 (49.6%) females were included at baseline. In multivariable model, lower meal consumption frequency was associated with increased BMI and BMI-for-age z-scores: children reporting <4 meals per day had a greater increase in BMI (β = 0.39; 95%CI: 0.17; 0.62) and BMI-for-age z-score (β = 0.07; 95%CI: 0.01; 0.13) compared to those reporting 5 meals per day. Results were consistent for those reporting exactly eating 4 times per day (β for BMI = 0.16; 95%CI: 0.02; 0.30, and β for BMI-for-age z-score = 0.05; 95%CI: 0.01; 0.09).

Conclusions: Children who eat <5 times per day, gain more BMI compared to those who eat ≥5 times. Parents should receive information to secure adequate nutrition for their children, both in terms of quality and quantity.

Keywords: Body mass index, meal frequency, longitudinal study, children, adolescent.

For peer review only

STRENGHTS AND LIMITATIONS OF THIS STUDY

- Results are supported by a population-based cohort of children with more than 9 years of follow-up in a middle income country. Our longitudinal analysis takes into account the repeated-measurements and including changes of both variables (outcome and exposure) over time.
- Recall bias can be an issue as mother's reports were used to evaluate the number of meals per day. In addition, other obesity markers, such as abdominal obesity (waist circumference), were not available for data analysis.
- Despite of the population-based nature of the study, selection bias may be a concern as 5% of the richest districts were excluded during the sampling process, potentially affecting the broad generalizability of our results.

For peer review only

INTRODUCTION

Current trends in children's and adolescent's body mass index (BMI) show a sustained and fast growth in different low- and middle-income countries (LMIC) (1). Moreover, the swift transition from underweight to overweight and obesity has also been reported in specific countries in Latin America, where the median of the prevalence distribution of excess bodyweight among school-aged children has been estimated in 29.8%, while this number was 17.8% among adolescents (2). Similar figures have been found in Peru among children between 6 and 9 years old (3).

Although changes in BMI may be explained by a misbalance between food intake and energy expenditure (4), some diet characteristics, such as diet quality and excess energy intake, may affect this association. In that sense, meal frequency has been described as a potential risk factor for BMI increase and, for instance, overweight and obesity (5).

Some studies have reported that regular (i.e. more frequent) meals are associated with lower risk of overweight and obesity (6-8), whereas other studies have not reported such association (9, 10). Moreover, a relatively recent systematic review and meta-analysis of 57 observational studies reported a significant relationship between frequent meals and better nutrition outcomes, including lower BMI (11). Nevertheless, this meta-analysis included only reports from high-income countries, showed correlations because most of the summarized studies were cross-sectional (only eight studies were longitudinal), and no studies were from Latin America. Moreover, among those longitudinal studies, the variation of frequency meal and BMI over time was not taken into account. Therefore, whether the association between meal frequency and BMI holds in Latin America, is unknown; though there could be differences because of the nutritional profiles and access to food patterns of these populations. This study aimed at assessing the association between meal frequency and BMI changes over time among children in Peru.

For peer review only

METHODS

Study design

This is a secondary analysis utilizing information of the Young Lives Study, a longitudinal ongoing prospective cohort conducted in four developing countries (Ethiopia, India, Peru and Vietnam) (12). The cohort started in 2002 and currently has five assessment rounds, i.e. a baseline and four follow-up evaluations.

Study participants

The Young Lives Study in Peru is comprised by two different cohorts: the younger cohort that initially enrolled 2052 children aged between 6 and 18 months, and the older cohort that included 1000 children between 7 and 8 years (12). For this work, we utilized information from the second (2006-07), third (2009-10), fourth (2013-14), and fifth (2016-17) rounds of the younger cohort in Peru as information about meal frequency was available in those years. As a result, data of children aged 4 to 5 years in the second round was used as our baseline, and information from the third, fourth and fifth rounds were included as follow-up assessments. Records with incomplete information in the variables of interest (meal frequency and body mass index) were excluded from the analysis.

Sampling

A sentinel site sampling strategy was used as previously published (13). Briefly, a multistage, cluster stratified, random sampling technique of sentinel sites was conducted. The initial sample frame was carried out at the district level, selecting 20 sentinel sites from a total of 1818 districts available. To oversampling poor areas, the top 5% richest districts were not included in the sampling process.

For peer review only

Maps of census tracts, comprising block of houses or set of houses, were used to randomly select one census tract in each district utilizing a random number table. All households in any given block or set of houses were visited to identify one household with at least one child meeting inclusion criteria. Different block or set of houses were approached until the total eligible households were completed. Three different teams, including fieldworkers, data-entry staff and supervisors were responsible for data collection and quality of six or seven sentinel sites.

Study variables

Two were the outcomes of interest for this study: body mass index (BMI) evaluated as numerical continuous variable and BMI-for-age z-score (14). We used these outcomes because we aimed at assessing changes over time using the information available from all the four study rounds. In addition, both definitions (i.e. BMI and BMI-for-age z-score) were used as BMI alone is not the best indicator of excess of weight among children under six years of age (14, 15).

The exposure of interest was meal frequency as reported by the parents based on seven questions related to the consumption of any food during the previous 24 hours before the interview. These seven questions were related to the moment in which the child ate any food (before breakfast, breakfast, between breakfast and midday meal, midday meal, between midday meal and evening meal, evening meal, and after evening meal). A simple addition of responses to these seven questions was used to estimate the total times the child ate in the last 24 hours. For analysis purposes, the variable was categorized as <4, 4, 5, 6, and 7 times per day (5), and the middle category (5 times per day) was used as the reference category. This process was conducted for each assessment included in the analysis (i.e. baseline and three follow-up evaluations).

Other variables were also included as potential confounders in regression models as evaluated at baseline: sex (boy or girl), age (in years), socioeconomic status assessed using a wealth index

For peer review only - http://bmjopen.bmj.com/site/about/guidelines.xhtml

created based on household assets and split in tertiles (low, middle and high), setting type (urban or rural), maternal and paternal education based on years of education (<7 years, 7-11 years, and ≥12 years), and maternal BMI as continuous variable, but also split according to traditional thresholds (normal [<25 kg/m^2], overweight [25-29 kg/m^2] and obese [≥30 kg/m^2]) (15).

Data analysis

STATA 13 for Windows (StataCorp, College Station, TX, US) was used for statistical analysis. Initially, the description of the study population was tabulated according to meal frequency using mean and standard deviation (SD) for numerical variables and proportions for categorical ones. Chi-squared test or analysis of variance was used for comparison between variables accordingly. Means and standard deviation (SD) of BMI and BMI-for-age z-score for baseline and each follow-up were calculated according to meal frequency at baseline (i.e. without taking into account correlation between measurements or any other variables). Finally, crude and adjusted linear mixed models with random intercepts were used to determine the association of interest (i.e. association between BMI or BMI-for-age z-score and meal frequency, accounting for the repeated-measure nature of the study and including changes of both variables over time). Three levels were fitted in the regression models (children assessment as level 1, subject as level 2, and sentinel site as level 3). BMI changes over time were presented as coefficients and their respective 95% confidence intervals (95% CI) controlling for different confounders.

Ethics

The Young Lives study was originally approved by the Ethics Committee Social Science Division, University of Oxford, UK, in 2006. In Peru, the approval was granted by the Research Ethics Committee of the Instituto de Investigacion Nutricional in Lima. Data is freely available under request and information is de-identified.

For peer review only

RESULTS

Baseline characteristics of the study population

A total of 2052 children were originally recruited in the younger cohort in Peru. Of them, 103 (5.0%) were lost to follow-up and 1 (0.1%) was excluded because of missing data (Figure 1). Thus, 1948 individuals were included in the baseline analysis, with a mean age of 4.3 (SD: 0.3) years and 966 (49.6%) were females. At baseline, meal frequency was on average 4.9 (SD: 1.0, range: 2 – 7) times per day and 847 (43.5%) reported eating 5 times per day, whereas 215 (11.0%) ate <4 times per day and 77 (4.0%) ate ≥7 times per day. The characteristics of the study population according to meal frequency at baseline are shown in Table 1. Of note, children age, maternal and paternal education, socioeconomic status, setting type, and maternal BMI were positively associated with meal frequency.

Meal frequency and body mass index during follow-up

Figure 1 shows in detail the number of participants in each of the follow-up assessments. On average, children were followed-up for a total of 9.6 (SD: 0.3) years. The proportion of children that reported eating ≥5 times per day dropped from 68.4% at baseline (4.3 ± 0.3 years of age) to 49.8% at the end of the follow-up (13.9 ± 0.1 years of age).

Table 2 shows a description of the mean BMI during each study follow-up rounds without taking into account intra-subject correlation. All the meal frequency groups showed an increase of BMI and BMI-for-age z-score, and this increase seemed to be higher in the group reporting a meal frequency of 5 times per day.

BMI and BMI-for-age z-score change over time

Results of the crude and adjusted regression models are shown in Table 3. According to multivariable model, our results show that lower frequency meal consumption was associated

For peer review only

with increased BMI and BMI-for-age z-scores. Children reporting <4 meals per day had a greater increase in BMI (β = 0.39; 95%CI: 0.17; 0.62) and BMI-for-age z-score (β = 0.07; 95%CI: 0.01; 0.13) compared to those reporting 5 meals per day. These results were similar for those reporting eating exactly 4 times per day (β for BMI = 0.16; 95%CI: 0.02; 0.30, and β for BMI-for-age z-score = 0.05; 95%CI: 0.01; 0.09); however, changes among those eating 6 or 7 times per day did not show association (See Figure 2).

For peer review only

DISCUSSION

Main findings

Our study shows evidence of an association between daily meal frequency and children's BMI changes over time. Although the magnitude of the association was small, less frequent meals were associated with lower BMI gain over time: those eating less than 5 times per day had greater BMI and BMI-for-age z-score measurements, whereas those eating ≥5 times per day did not have an increase in these outcomes. In addition, almost two thirds of the study population reported eating ≥5 times per day at baseline (i.e. when the child aged between 4-5 years), but this number reduced to less than half of participants when children were between 14 and 15 years.

Comparison with previous studies

Two systematic reviews have approximated to the association proposed in this paper. The first one reported inconsistent and weak evidence of an inverse association between meal frequency and risk of childhood overweight (16). This review used 15 reports and only 4 were longitudinal studies. The second systematic review, conducted in 2017, found a significant relationship between frequent meals and better nutritional health using 57 studies, both in younger and older children, across countries and socioeconomic groups (11). However, the latter review only

reported weighted mean correlations and no risk of overnutrition or changes of BMI over time, and many of the summarized reports studied convenience samples. Our work agrees with the current literature and adds evidence from new populations (children in Latin America) and followed a strong observational design (prospective cohort).

Many of the studies looking for the association of interest have been cross-sectional, and have used outcomes based on overweight or obesity. Toschke et al reported a decrease in the prevalence of obesity by number of daily meals in two different studies (5, 8); whereas in a cross-sectional assessment of a Finnish cohort study of adolescents they reported that adolescents who ate five meals/day were at lower risk for general and abdominal obesity as well as hypertriglyceridemia (6). Thus, our study expands on previous findings by using repeated measurements following appropriate longitudinal analysis to verify the association of interest in a population-based cohort study of children transitioning to adolescents.

Of note, our findings support the fact that the number of meals per day seems to decrease from childhood to adolescence. Therefore, children may lose the beneficial effects of eating more often throughout the day, thus increasing the probability of having overnutrition during adolescence.

Public health relevance

Our data suggest that a higher number of daily meals may reduce body mass index over time, reducing potentially the risk of overweight and obesity in children and adolescents. Effects of meal frequency on endocrine responses and regulation might explain our findings. Increased meal frequency may attenuate postprandial metabolic and endocrine responses to nutrient intake, with the subsequent reduction in insulin secretion (7, 17).

For peer review only

Since there is more evidence that skipping meals, especially breakfast, is associated with increasing risk of overnutrition (i.e. overweight and obesity) among children (18), the promotion of a regular meal pattern including at least 5 meals per day should be guaranteed. In addition, the quality and adequate composition of meals must be also promoted to children and, especially, their parents, families and caregivers.

Strength and limitations

Our findings are supported by a population-based cohort of children with more than 9 years of follow-up in a middle income country. Moreover, our longitudinal analysis shows strong association between meal frequency and BMI, taking into account the repeated-measurements and including changes of both variables over time. However, this study has limitations that should be highlighted. First, mother's reports were used to evaluate the number of meals per day based on the last day before the interview; recall bias may arise as a concern. Nevertheless, our results are congruent with previous studies (19-21). Second, other obesity markers, such as abdominal obesity, were not available for data analysis. Third, no all the potential confounders were available to adjust our regression models (e.g. physical activity or portion size); some residual confounding may be present. Finally, selection bias could be a concern as 5% of the richest districts were excluded during the sampling process, potentially affecting the broad generalizability of our results. Moreover, in countries undergoing nutrition transition such as Peru, changes in BMI are initially seen in the wealthiest individuals (22). Nonetheless, our estimates are informative for the general population though should be interpreted cautiously for families with high socio-economic status.

Conclusion

For peer review only

There is evidence of association between the number of meals per day consumed by a child and increasing BMI over time: those who ate less than 5 times per day had greater BMI and BMI-for-age z-score measurements compared to those eating ≥5 times per day. The promotion of a regular meal pattern including at least 5 meals per day should be guaranteed.

FUNDING STATEMENT

This research received no specific grant from any funding agency in the public, commercial or not-for-profit sectors. Rodrigo M Carrillo-Larco is supported by a Wellcome Trust International Training Fellowship (214185/Z/18/Z).

COMPETING INTEREST STATEMENT

The authors declare that no competing interests exist.

PATIENT AND PUBLIC INVOLVEMENT

Patients or the public were not involved in the design, or conduct, or reporting, or dissemination plans of our research.

AUTHORS' CONTRIBUTION

AB-O and RMC-L conceived the idea. RMC-L conducted the data cleaning and harmonization. AB-O conducted the statistical analysis. AB-O wrote the first version of the manuscript with input from RMC-L. Both authors approved the submitted version.

DATA SHARING STATEMENT

For peer review only

Data is available under request via the Young Lives webpage (https://www.younglives.org.uk/). Statistical code is available at: doi.org/10.6084/m9.figshare.11629092 (STATA syntax).

REFERENCES

1. NCD Risk Factor Collaboration (NCD-RisC). Worldwide trends in body-mass index, underweight, overweight, and obesity from 1975 to 2016: a pooled analysis of 2416 population-based measurement studies in 128.9 million children, adolescents, and adults. Lancet (London, England). 2017;390(10113):2627-42.
2. Rivera JA, de Cossio TG, Pedraza LS, Aburto TC, Sanchez TG, Martorell R. Childhood and adolescent overweight and obesity in Latin America: a systematic review. The lancet Diabetes & endocrinology. 2014;2(4):321-32.
3. Pajuelo-Ramirez J, Sanchez-Abanto J, Alvarez-Dongo D, Tarqui-Mamani C, Aguero-Zamora R. [Overweight, obesity and chronic mal nutrition in 6 to 9 year-old children in Peru, 2009-2010]. Revista peruana de medicina experimental y salud publica. 2013;30(4):583-9.
4. Romieu I, Dossus L, Barquera S, Blottiere HM, Franks PW, Gunter M, et al. Energy balance and obesity: what are the main drivers? Cancer causes & control : CCC. 2017;28(3):247-58.
5. Toschke AM, Kuchenhoff H, Koletzko B, von Kries R. Meal frequency and childhood obesity. Obesity research. 2005;13(11):1932-8.
6. Jaaskelainen A, Schwab U, Kolehmainen M, Pirkola J, Jarvelin MR, Laitinen J. Associations of meal frequency and breakfast with obesity and metabolic syndrome traits in adolescents of Northern Finland Birth Cohort 1986. Nutrition, metabolism, and cardiovascular diseases : NMCD. 2013;23(10):1002-9.

For peer review only

7. Koletzko B, Toschke AM. Meal patterns and frequencies: do they affect body weight in children and adolescents? Critical reviews in food science and nutrition. 2010;50(2):100-5.

8. Toschke AM, Thorsteinsdottir KH, von Kries R. Meal frequency, breakfast consumption and childhood obesity. International journal of pediatric obesity : IJPO : an official journal of the International Association for the Study of Obesity. 2009;4(4):242-8.

9. Lee SY, Ha SA, Seo JS, Sohn CM, Park HR, Kim KW. Eating habits and eating behaviors by family dinner frequency in the lower-grade elementary school students. Nutrition research and practice. 2014;8(6):679-87.

10. Mamun AA, Lawlor DA, O'Callaghan MJ, Williams GM, Najman JM. Positive maternal attitude to the family eating together decreases the risk of adolescent overweight. Obesity research. 2005;13(8):1422-30.

11. Dallacker M, Hertwig R, Mata J. The frequency of family meals and nutritional health in children: a meta-analysis. Obesity reviews : an official journal of the International Association for the Study of Obesity. 2018;19(5):638-53.

12. Barnett I, Ariana P, Petrou S, Penny ME, Duc le T, Galab S, et al. Cohort profile: the Young Lives study. International journal of epidemiology. 2013;42(3):701-8.

13. Escobal J, Flores E. An Assessment of the Young Lives Sampling Approach in Peru. Technical Note 3. Oxford, United Kingdom: Young Lives, 2008.

14. Must A, Anderson SE. Body mass index in children and adolescents: considerations for population-based applications. International journal of obesity (2005). 2006;30(4):590-4.

15. World Health Organization. Obesity and overweight: WHO; 2018 [cited 2020 January 7]. Available from: https://www.who.int/news-room/fact-sheets/detail/obesity-and-overweight.

For peer review only

16. Valdes J, Rodriguez-Artalejo F, Aguilar L, Jaen-Casquero MB, Royo-Bordonada MA. Frequency of family meals and childhood overweight: a systematic review. Pediatric obesity. 2013;8(1):e1-e13.

17. Jenkins DJ, Khan A, Jenkins AL, Illingworth R, Pappu AS, Wolever TM, et al. Effect of nibbling versus gorging on cardiovascular risk factors: serum uric acid and blood lipids. Metabolism: clinical and experimental. 1995;44(4):549-55.

18. Ardeshirlarijani E, Namazi N, Jabbari M, Zeinali M, Gerami H, Jalili RB, et al. The link between breakfast skipping and overweigh/obesity in children and adolescents: a meta-analysis of observational studies. Journal of diabetes and metabolic disorders. 2019;18(2):657-64.

19. Gable S, Chang Y, Krull JL. Television watching and frequency of family meals are predictive of overweight onset and persistence in a national sample of school-aged children. Journal of the American Dietetic Association. 2007;107(1):53-61.

20. Laurson K, Eisenmann JC, Moore S. Lack of association between television viewing, soft drinks, physical activity and body mass index in children. Acta paediatrica (Oslo, Norway : 1992). 2008;97(6):795-800.

21. Taveras EM, Rifas-Shiman SL, Berkey CS, Rockett HR, Field AE, Frazier AL, et al. Family dinner and adolescent overweight. Obesity research. 2005;13(5):900-6.

22. Popkin BM, Adair LS, Ng SW. Global nutrition transition and the pandemic of obesity in developing countries. Nutrition reviews. 2012;70(1):3-21.

For peer review only

TABLES AND FIGURES

Figure 1: Flowchart of participants analyzed using the Young Lives younger cohort

For peer review only

Figure 2: Variation over time in (A) BMI and (B) BMI-for-age z-score according to meal frequency controlling for potential confounders

(A) (B)

For peer review only

Table 1: Characteristics of the study population according to meal frequency at baseline

	Meal frequency at baseline (times per day)					
	< 4 (n = 217)	4 (n = 402)	5 (n = 851)	6 (n = 409)	7 (n = 77)	p-value*
**Sex, n (%)**						0.52
Female	102 (47.0%)	201 (50.0%)	438 (51.5%)	191 (46.7%)	39 (50.7%)	
Male	115 (53.0%)	201 (50.0%)	413 (48.5%)	218 (53.3%)	38 (49.4%)	
**Age, mean (SD)**						< 0.001
In years	4.2 (0.2)	4.3 (0.3)	4.4 (0.3)	4.3 (0.3)	4.3 (0.2)	
**Maternal education, n (%)**						< 0.001
< 7 years	161 (74.5%)	219 (54.9%)	312 (37.1%)	150 (36.7%)	28 (36.8%)	
7 – 11 years	47 (21.8%)	139 (34.8%)	340 (40.4%)	158 (38.6%)	28 (36.8%)	
12+ years	8 (3.7%)	41 (10.3%)	189 (22.5%)	101 (24.7%)	20 (26.3%)	
**Paternal education, n (%)**						< 0.001
< 7 years	116 (56.0%)	149 (38.3%)	248 (30.0%)	112 (28.3%)	24 (32.0%)	
7 – 11 years	80 (38.7%)	181 (46.5%)	369 (44.6%)	185 (46.7%)	27 (36.0%)	
12+ years	11 (5.3%)	59 (15.2%)	211 (25.4%)	99 (25.0%)	24 (32.0%)	
**Socioeconomic status, n (%)**						< 0.001
Low	144 (66.3%)	167 (41.6%)	219 (25.7%)	100 (24.5%)	24 (31.2%)	
Middle	57 (26.3%)	148 (36.8%)	283 (33.3%)	140 (34.2%)	25 (32.5%)	
High	16 (7.4%)	87 (21.6%)	349 (41.0%)	169 (41.3%)	28 (36.4%)	
**Setting type, n (%)**						< 0.001
Urban	53 (24.4%)	195 (48.5%)	544 (63.9%)	248 (60.6%)	42 (54.6%)	
Rural	164 (75.6%)	207 (51.5%)	307 (36.1%)	161 (39.4%)	35 (45.4%)	
**Maternal BMI, mean (SD)**						0.02
In Kg/m^2	25.3 (3.3)	25.9 (4.1)	26.4 (4.5)	26.1 (3.8)	25.8 (3.7)	
**Maternal BMI, n (%)**						0.10

For peer review only

Normal	108 (52.2%)	175 (46.1%)	343 (42.6%)	171 (43.4%)	33 (46.5%)
Overweight	84 (40.6%)	148 (38.9%)	333 (41.4%)	162 (41.1%)	29 (40.8%)
Obese	15 (7.2%)	57 (15.0%)	129 (16.0%)	61 (15.5%)	9 (12.7%)

SD = standard deviation; BMI = Body mass index
* Comparisons were conducted using Chi-squared tests for categorical variables, and analysis of variance for numerical ones.

For peer review only

Table 2: BMI and BMI z-score by meal frequency at baseline: Mean and standard deviation (SD) during follow-up

	**Mean and standard deviation of BMI and BMI z-score**			
	**Baseline (n = 1948)**	**First follow-up (n = 1904)**	**Second follow-up (n = 1850)**	**Third follow-up (n = 1810)**
**Meal frequency**	**BMI (in Kg/m²): mean (SD)**			
< 4 times per day	16.4 (1.9)	16.4 (1.7)	18.5 (2.5)	21.1 (2.7)
4 times per day	16.4 (1.5)	16.8 (2.1)	19.4 (3.0)	21.7 (3.3)
5 times per day	16.5 (1.9)	17.0 (2.4)	19.9 (3.4)	21.8 (3.4)
6 times per day	16.3 (1.6)	16.9 (2.4)	19.8 (3.3)	21.6 (3.4)
7 times per day	16.5 (2.4)	16.8 (2.4)	19.4 (3.3)	21.0 (2.9)
**Meal frequency**	**BMI z-score: mean (SD)**			
< 4 times per day	0.67 (1.06)	0.29 (0.92)	0.18 (0.92)	0.29 (0.81)
4 times per day	0.68 (0.92)	0.49 (1.02)	0.50 (1.02)	0.44 (0.93)
5 times per day	0.71 (1.09)	0.59 (1.08)	0.64 (1.11)	0.44 (1.01)
6 times per day	0.65 (0.98)	0.55 (1.13)	0.61 (1.08)	0.41 (0.99)
7 times per day	0.72 (1.33)	0.42 (1.14)	0.45 (1.15)	0.26 (0.90)

SD = Standard deviation; BMI = body mass index

For peer review only

Table 3: Meal frequency and changes over time of body mass index: crude and adjusted linear mixed models

	Crude model Coefficient (95% CI)	Adjusted model* Coefficient (95% CI)
**Meal frequency**	**Change in BMI (Kg/m²)**	
< 4 times per day	**1.18 (0.73; 1.64)**	**0.39 (0.17; 0.62)**
4 times per day	**0.71 (0.43; 0.98)**	**0.16 (0.02; 0.30)**
5 times per day	1 (Reference)	1 (Reference)
6 times per day	**-0.54 (-0.68; -0.40)**	-0.03 (-0.15; 0.09)
7 times per day	**-0.73 (-1.26; -0.21)**	0.08 (-0.33; 0.50)
**Meal frequency**	**Change in BMI z-score**	
< 4 times per day	0.01 (-0.08; 0.10)	**0.07 (0.01; 0.13)**
4 times per day	0.01 (-0.03; 0.06)	**0.05 (0.01; 0.09)**
5 times per day	1 (Reference)	1 (Reference)
6 times per day	0.01 (-0.03; 0.06)	-0.01 (-0.05; 0.04)
7 times per day	0.06 (-0.10; 0.21)	0.04 (-0.13; 0.20)

95% CI: 95% confidence intervals; BMI = body mass index
* Adjusted for sex, age, maternal education, paternal education, wealth index, setting type, and maternal BMI (in categories). Estimates in bold are statistical significant.

For peer review only

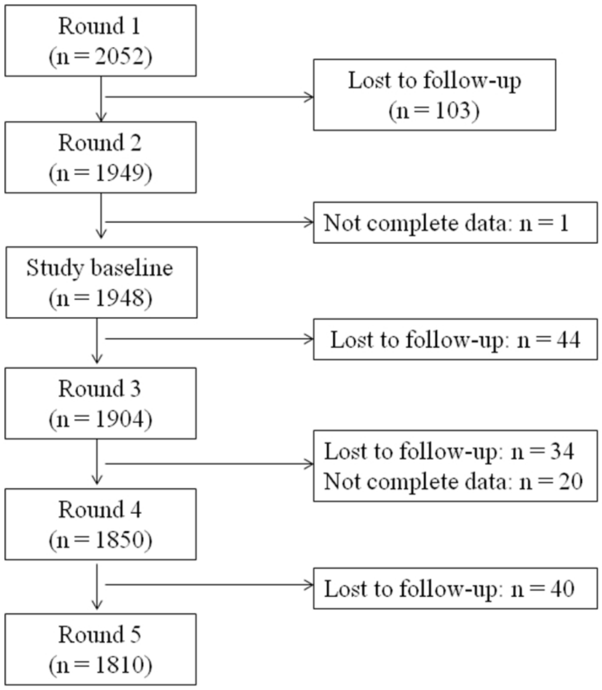

Figure 1: Flowchart of participants

150x112mm (300 x 300 DPI)

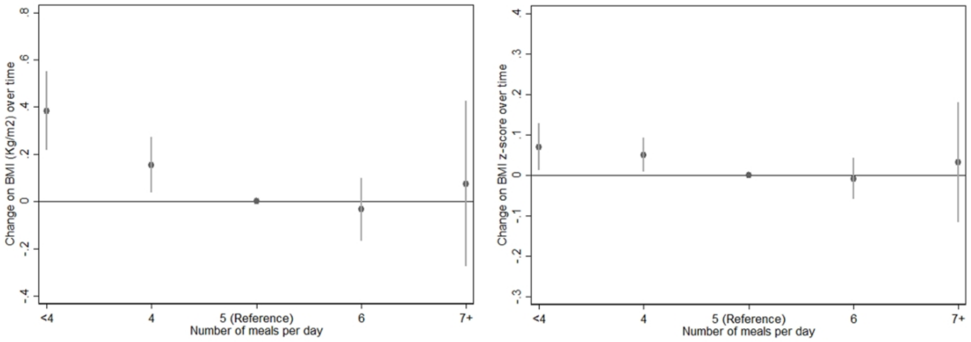

Figure 2: Changes of BMI and BMI-for-age z-score over time according to meal frequency

250x92mm (300 x 300 DPI)

STROBE 2007 (v4) Statement—Checklist of items that should be included in reports of *cohort studies*

Section/Topic	Item #	Recommendation	Reported on page #
**Title and abstract**	1	(*a*) Indicate the study's design with a commonly used term in the title or the abstract	1
		(*b*) Provide in the abstract an informative and balanced summary of what was done and what was found	3
**Introduction**			
Background/rationale	2	Explain the scientific background and rationale for the investigation being reported	6
Objectives	3	State specific objectives, including any prespecified hypotheses	6
**Methods**			
Study design	4	Present key elements of study design early in the paper	7
Setting	5	Describe the setting, locations, and relevant dates, including periods of recruitment, exposure, follow-up, and data collection	7
Participants	6	(*a*) Give the eligibility criteria, and the sources and methods of selection of participants. Describe methods of follow-up	7
		(*b*) For matched studies, give matching criteria and number of exposed and unexposed	
Variables	7	Clearly define all outcomes, exposures, predictors, potential confounders, and effect modifiers. Give diagnostic criteria, if applicable	8
Data sources/ measurement	8*	For each variable of interest, give sources of data and details of methods of assessment (measurement). Describe comparability of assessment methods if there is more than one group	8
Bias	9	Describe any efforts to address potential sources of bias	9
Study size	10	Explain how the study size was arrived at	9
Quantitative variables	11	Explain how quantitative variables were handled in the analyses. If applicable, describe which groupings were chosen and why	9
Statistical methods	12	(*a*) Describe all statistical methods, including those used to control for confounding	9
		(*b*) Describe any methods used to examine subgroups and interactions	9
		(*c*) Explain how missing data were addressed	NA
		(*d*) If applicable, explain how loss to follow-up was addressed	NA
		(*e*) Describe any sensitivity analyses	NA
**Results**			

For peer review only

Participants	13*	(a) Report numbers of individuals at each stage of study—eg numbers potentially eligible, examined for eligibility, confirmed eligible, included in the study, completing follow-up, and analysed	10
		(b) Give reasons for non-participation at each stage	10
		(c) Consider use of a flow diagram	Figure 1
Descriptive data	14*	(a) Give characteristics of study participants (eg demographic, clinical, social) and information on exposures and potential confounders	10
		(b) Indicate number of participants with missing data for each variable of interest	Figure 1
		(c) Summarise follow-up time (eg, average and total amount)	10
Outcome data	15*	Report numbers of outcome events or summary measures over time	10
Main results	16	(*a*) Give unadjusted estimates and, if applicable, confounder-adjusted estimates and their precision (eg, 95% confidence interval). Make clear which confounders were adjusted for and why they were included	10-11
		(*b*) Report category boundaries when continuous variables were categorized	10-11
		(*c*) If relevant, consider translating estimates of relative risk into absolute risk for a meaningful time period	10-11
Other analyses	17	Report other analyses done—eg analyses of subgroups and interactions, and sensitivity analyses	10-11
**Discussion**			
Key results	18	Summarise key results with reference to study objectives	11
**Limitations**			
Interpretation	20	Give a cautious overall interpretation of results considering objectives, limitations, multiplicity of analyses, results from similar studies, and other relevant evidence	11-12
Generalisability	21	Discuss the generalisability (external validity) of the study results	11-12
**Other information**			
Funding	22	Give the source of funding and the role of the funders for the present study and, if applicable, for the original study on which the present article is based	13

*Give information separately for cases and controls in case-control studies and, if applicable, for exposed and unexposed groups in cohort and cross-sectional studies.

Note: An Explanation and Elaboration article discusses each checklist item and gives methodological background and published examples of transparent reporting. The STROBE checklist is best used in conjunction with this article (freely available on the Web sites of PLoS Medicine at http://www.plosmedicine.org/, Annals of Internal Medicine at http://www.annals.org/, and Epidemiology at http://www.epidem.com/). Information on the STROBE Initiative is available at www.strobe-statement.org.

For peer review only

BMJ Open

Longitudinal association between food frequency and changes in body mass index: a prospective cohort study

Journal:	*BMJ Open*
Manuscript ID	bmjopen-2020-037057.R1
Article Type:	Original research
Date Submitted by the Author:	28-Feb-2020
Complete List of Authors:	Bernabe-Ortiz, Antonio; Universidad Peruana Cayetano Heredia, CRONICAS Centre of Excellence in Chronic Diseases<br>Carrillo-Larco, Rodrigo; Universidad Peruana Cayetano Heredia, CRONICAS Center of Excellence; CRONICAS Center of Excellence in Chronic Diseases
**Primary Subject Heading**:	Nutrition and metabolism
Secondary Subject Heading:	Epidemiology, Public health
Keywords:	EPIDEMIOLOGY, NUTRITION & DIETETICS, PUBLIC HEALTH

SCHOLARONE™
Manuscripts

I, the Submitting Author has the right to grant and does grant on behalf of all authors of the Work (as defined in the below author licence), an exclusive licence and/or a non-exclusive licence for contributions from authors who are: i) UK Crown employees; ii) where BMJ has agreed a CC-BY licence shall apply, and/or iii) in accordance with the terms applicable for US Federal Government officers or employees acting as part of their official duties; on a worldwide, perpetual, irrevocable, royalty-free basis to BMJ Publishing Group Ltd ("BMJ") its licensees and where the relevant Journal is co-owned by BMJ to the co-owners of the Journal, to publish the Work in this journal and any other BMJ products and to exploit all rights, as set out in our licence.

The Submitting Author accepts and understands that any supply made under these terms is made by BMJ to the Submitting Author unless you are acting as an employee on behalf of your employer or a postgraduate student of an affiliated institution which is paying any applicable article publishing charge ("APC") for Open Access articles. Where the Submitting Author wishes to make the Work available on an Open Access basis (and intends to pay the relevant APC), the terms of reuse of such Open Access shall be governed by a Creative Commons licence – details of these licences and which Creative Commons licence will apply to this Work are set out in our licence referred to above.

Other than as permitted in any relevant BMJ Author's Self Archiving Policies, I confirm this Work has not been accepted for publication elsewhere, is not being considered for publication elsewhere and does not duplicate material already published. I confirm all authors consent to publication of this Work and authorise the granting of this licence.

For peer review only

Longitudinal association between food frequency and changes in body mass index: a prospective cohort study

Antonio Bernabe-Ortiz, MD, MPH, PhD[1,2]; Rodrigo M. Carrillo-Larco, MD[1,3,4]

1. CRONICAS Center of Excellence in Chronic Diseases, Universidad Peruana Cayetano Heredia, Lima, Peru
2. Universidad Cientifica del Sur, Lima, Perú
3. Department of Epidemiology and Biostatistics, School of Public Health, Imperial College London, London, UK
4. Centro de Estudios de Población, Universidad Católica los Ángeles de Chimbote (ULADECH-Católica), Chimbote, Peru

For peer review only

Short title:

Meal frequency and body mass index

E-mails:

Antonio Bernabe-Ortiz: Antonio.Bernabe@upch.pe

Rodrigo M. Carrillo-Larco: Rodrigo.Carrillo@upch.pe

Corresponding autor:

Antonio Bernabe-Ortiz, MD, MPH, PhD

CRONICAS Center of Excellence in Chronic Diseases, Universidad Peruana Cayetano Heredia

Av. Armendariz 445, Miraflores, Lima, Peru

E-mail: Antonio.Bernabe@upch.pe

Phone: 51-1-241-6978

Word count:

Abstract: 227 words

Manuscript (from Introduction to References): 3000 words (total: 4318 words)

For peer review only

ABSTRACT

Objectives: Analyzing data of the Young Lives Study in Peru, we aimed at assessing the association between daily meal frequency and BMI changes between 2006 and 2016.

Design: Secondary analysis of a prospective ongoing cohort study.

Setting: 20 sentinel sites in Peru

Participants: Children enrolled in the younger cohort of the Young Lives Study. We used information from the second (2006-07), third (2009-10), fourth (2013-14), and fifth (2016-17) rounds of the younger cohort in Peru.

Primary and secondary outcomes: Body mass index (BMI) as well as BMI-for-age z-score, both as numerical variables.

Results: Data from 1948 children, mean age 4.3 (SD: 0.3) years and 966 (49.6%) females were included at baseline. In multivariable model, lower meal consumption frequency was associated with increased BMI and BMI-for-age z-scores: children reporting <4 meals per day had a greater increase in BMI ($\beta = 0.39$; 95%CI: 0.17; 0.62) and BMI-for-age z-score ($\beta = 0.07$; 95%CI: 0.01; 0.13) compared to those reporting 5 meals per day. Results were consistent for those reporting exactly eating 4 times per day (β for BMI = 0.16; 95%CI: 0.02; 0.30, and β for BMI-for-age z-score = 0.05; 95%CI: 0.01; 0.09).

Conclusions: Children who eat <5 times per day, gain more BMI compared to those who eat ≥5 times. Parents should receive information to secure adequate nutrition for their children, both in terms of quality and quantity.

Keywords: Body mass index, meal frequency, longitudinal study, children, adolescent.

For peer review only

STRENGHTS AND LIMITATIONS OF THIS STUDY

- Results are supported by a population-based cohort of children with more than nine years of follow-up in a middle income country.
- Our analysis took into account the repeated-measure nature of the data including changes of both outcome and exposure over time.
- Recall bias can be an issue as mother’s reports were used to evaluate the number of meals per day.

For peer review only

INTRODUCTION

Current trends in children's and adolescent's body mass index (BMI) show a sustained and fast growth in different low- and middle-income countries (LMIC) (1). Moreover, the swift transition from underweight to overweight and obesity has also been reported in specific countries in Latin America, where the median of the prevalence distribution of excess bodyweight among school-aged children has been estimated in 29.8%, while this number was 17.8% among adolescents (2). Similar figures have been found in Peru among children between 6 and 9 years old (3).

Changes in BMI are explained by a misbalance between food intake and energy expenditure (4); therefore, some diet characteristics, such as diet quality and excess energy intake, may affect these changes. In that sense, meal frequency has been described as a potential risk factor for BMI increase and, for instance, overweight and obesity (5).

Some studies have reported that regular (i.e. more frequent) meals are associated with lower risk of overweight and obesity (6-8), whereas other studies have not reported such association (9, 10). Moreover, a relatively recent systematic review and meta-analysis of 57 observational studies reported a significant relationship between frequent meals and better nutrition outcomes, including lower BMI (11). Nevertheless, this meta-analysis included only reports from high-income countries, showed correlations because most of the summarized studies were cross-sectional (only eight studies were longitudinal), and only one study was from Latin America (Puerto Rico) (12). Moreover, among those longitudinal studies, the variation of frequency meal and BMI over time was not taken into account. Therefore, whether the association between meal frequency and BMI holds in Latin America, is unknown; though there could be differences because of the nutritional profiles and access to food patterns of these populations. For example, food insecurity is high in Peru, especially in rural and poor areas (13), and households with an

For peer review only

insecure profile usually have a poor diet quality and consume more carbohydrates (14). Thus, this study aimed at assessing the association between meal frequency and BMI changes over time among children in Peru. We hypothesized that children eating less frequently will have a greater gain of BMI over time.

METHODS

Study design

This is a secondary analysis utilizing information of the Young Lives Study, a longitudinal ongoing prospective cohort conducted in four developing countries (Ethiopia, India, Peru and Vietnam) (15). The cohort started in 2002 and currently has five assessment rounds, i.e. a baseline and four follow-up evaluations.

Study participants

The Young Lives Study in Peru is comprised by two different cohorts: the younger cohort that initially enrolled 2052 children aged between 6 and 18 months, and the older cohort that included 1000 children between 7 and 8 years (15). For this work, we utilized information from the second (2006-07), third (2009-10), fourth (2013-14), and fifth (2016-17) rounds of the younger cohort in Peru as information about meal frequency was available in that cohort. As a result, information of the second round was used as the baseline of the cohort, and information from the third, fourth and fifth rounds were included as follow-up assessments. Records with incomplete information in the variables of interest (meal frequency and body mass index) were excluded from the analysis.

Sampling

For peer review only

A sentinel site sampling strategy was used as previously published (16). Briefly, a multistage, cluster stratified, random sampling technique of sentinel sites was conducted. The initial sample frame was carried out at the district level, selecting 20 sentinel sites from a total of 1818 districts available. To oversampling poor areas, the top 5% richest districts were not included in the sampling process.

Maps of census tracts, comprising block of houses or set of houses, were used to randomly select one census tract in each district utilizing a random number table. All households in any given block or set of houses were visited to identify one household with at least one child meeting inclusion criteria. Different block or set of houses were approached until the total eligible households were completed. Three different teams, including fieldworkers, data-entry staff and supervisors were responsible for data collection and quality of six or seven sentinel sites.

Study variables

Two were the outcomes of interest for this study: body mass index (BMI) evaluated as numerical continuous variable and BMI-for-age z-score. Both height and weight were measured in each children assessment and used to estimate BMI as usual. BMI-for-age z-score was based on the World Health Organization reference (17). We used these outcomes because we aimed at assessing changes over time using the information available from all the four study rounds. In addition, both definitions (i.e. BMI and BMI-for-age z-score) were used as BMI alone is not the best indicator of excess of weight among children under six years of age (17, 18).

The exposure of interest was meal frequency as reported by the parents based on seven questions related to the consumption of any possible meal or snack during the previous 24 hours before the interview. The question was asked as follow: “During the previous 24-hour period, did the child consume: any food before a morning meal (breakfast)? Any morning meal (breakfast)? Any food

For peer review only - http://bmjopen.bmj.com/site/about/guidelines.xhtml

between morning and midday meals? Any midday meal? Any food between midday and evening meals? Any evening meal? Any food after the evening meal? A simple addition of yes/no responses to these seven questions was used to estimate the total number of meals the child ate in the last 24 hours. For analysis purposes, the variable was categorized as <4, 4, 5, 6, and 7 times per day (5), and the middle category (5 times per day) was used as the reference category. This process was conducted for each assessment included in the analysis (i.e. baseline and three follow-up evaluations).

Other variables were also included as potential confounders in regression models as evaluated at baseline: sex (boy or girl), age (in years), socioeconomic status assessed using a wealth index created based on household assets and split in tertiles (low, middle and high), setting type (urban or rural), maternal and paternal education based on years of education (<7 years, 7-11 years, and ≥12 years), and maternal BMI as continuous variable, but also split according to traditional thresholds (normal [<25 kg/m^2], overweight [25-29 kg/m^2] and obese [≥30 kg/m^2]) (18).

Data analysis

STATA 13 for Windows (StataCorp, College Station, TX, US) was used for statistical analysis. Initially, the description of the study population was tabulated according to meal frequency using mean and standard deviation (SD) for numerical variables and proportions for categorical ones. Chi-squared test or analysis of variance was used for comparison between variables accordingly. Means and standard deviation (SD) of BMI and BMI-for-age z-score for baseline and each follow-up were calculated according to meal frequency at baseline (i.e. without taking into account correlation between measurements or any other variables). Finally, crude and adjusted linear mixed models with random intercepts were used to determine the association of interest (i.e. association between BMI or BMI-for-age z-score and meal frequency, accounting for the

For peer review only

repeated-measure nature of the study and including changes of both variables over time). Three levels were fitted in the regression models (children assessment as level 1, subject as level 2, and sentinel site as level 3). BMI changes over time were presented as coefficients and their respective 95% confidence intervals (95% CI) controlling for different confounders.

Ethics

The Young Lives study was originally approved by the Ethics Committee Social Science Division, University of Oxford, UK, in 2002. In Peru, the approval was granted by the Research Ethics Committee of the Instituto de Investigacion Nutricional in Lima. Data is freely available under request and information is de-identified.

RESULTS

Baseline characteristics of the study population

A total of 2052 children were originally recruited in the younger cohort in Peru. Of them, 103 (5.0%) were lost to follow-up and 1 (0.1%) was excluded because of missing data (Figure 1). Thus, 1948 individuals were included in the baseline analysis, with a mean age of 4.3 (SD: 0.3) years and 966 (49.6%) were females. At baseline, meal frequency was on average 4.9 (SD: 1.0, range: 2 – 7) times per day and 847 (43.5%) reported eating 5 times per day, whereas 215 (11.0%) ate <4 times per day and 77 (4.0%) ate ≥7 times per day. The characteristics of the study population according to meal frequency at baseline are shown in Table 1. Of note, children age, maternal and paternal education, socioeconomic status, setting type, and maternal BMI were positively associated with meal frequency.

Meal frequency and body mass index during follow-up

For peer review only

Figure 1 shows in detail the number of participants in each of the follow-up assessments. On average, children were followed-up for a total of 9.6 (SD: 0.3) years. The proportion of children that reported eating ≥5 times per day dropped from 68.4% at baseline (4.3 ± 0.3 years of age) to 49.8% at the end of the follow-up (13.9 ± 0.1 years of age).

Table 2 shows a description of the mean BMI during each study follow-up rounds without taking into account intra-subject correlation. All the meal frequency groups showed an increase of BMI and BMI-for-age z-score, and this increase seemed to be higher in the group reporting a meal frequency of 5 times per day.

BMI and BMI-for-age z-score change over time

Results of the crude and adjusted regression models are shown in Table 3. According to multivariable model, our results show that lower frequency meal consumption was associated with increased BMI and BMI-for-age z-scores. Children reporting <4 meals per day had a greater increase in BMI (β = 0.39; 95%CI: 0.17; 0.62) and BMI-for-age z-score (β = 0.07; 95%CI: 0.01; 0.13) compared to those reporting 5 meals per day. These results were similar for those reporting eating exactly 4 times per day (β for BMI = 0.16; 95%CI: 0.02; 0.30, and β for BMI-for-age z-score = 0.05; 95%CI: 0.01; 0.09); however, changes among those eating 6 or 7 times per day did not show association (See Figure 2).

DISCUSSION

Main findings

Our study shows evidence of an association between daily meal frequency and children's BMI changes over time. Although the magnitude of the association was small, less frequent meals were associated with greater BMI gain over time: those eating less than 5 times per day had

For peer review only

greater BMI and BMI-for-age z-score measurements, whereas those eating ≥5 times per day did not have an increase in these outcomes. In addition, almost two thirds of the study population reported eating ≥5 times per day at baseline (i.e. when the child aged between 4-5 years), but this number reduced to less than half of participants when children were between 14 and 15 years.

Comparison with previous studies

Two systematic reviews have approximated to the association proposed in this paper. The first one reported inconsistent and weak evidence of an inverse association between meal frequency and risk of childhood overweight (19). This review used 15 reports and only 4 were longitudinal studies. The second systematic review, conducted in 2018, found a significant relationship between frequent meals and better nutritional health using 57 studies, both in younger and older children, across countries and socioeconomic groups (11). However, the latter review only reported weighted mean correlations and no risk of overnutrition or changes of BMI over time, and many of the summarized reports studied convenience samples. Our work agrees with the current literature and adds more evidence from new populations (children in Latin America as limited information is available) and followed a strong observational design (prospective cohort). Many of the studies looking for the association of interest have been cross-sectional, and have used outcomes based on overweight or obesity. Toschke et al reported a decrease in the prevalence of obesity by number of daily meals in two different studies (5, 8); whereas in a cross-sectional assessment of a Finnish cohort study of adolescents they reported that adolescents who ate five meals/day were at lower risk for general and abdominal obesity as well as hypertriglyceridemia (6). Thus, our study expands on previous findings by using repeated measurements following appropriate longitudinal analysis to verify the association of interest in a population-based cohort study of children transitioning to adolescents.

For peer review only

Of note, our findings support the fact that the number of meals per day seems to decrease from childhood to adolescence. Therefore, children may lose the beneficial effects of eating more often throughout the day, thus increasing the probability of having overnutrition during adolescence.

Public health relevance

Our data suggest that a higher number of daily meals may reduce body mass index over time, reducing potentially the risk of overweight and obesity in children and adolescents. Effects of meal frequency on endocrine responses and regulation might explain our findings. Increased meal frequency may attenuate postprandial metabolic and endocrine responses to nutrient intake, with the subsequent reduction in insulin secretion (7, 20).

Since there is more evidence that skipping meals, especially breakfast, is associated with increasing risk of overnutrition (i.e. overweight and obesity) among children (21), the promotion of a regular meal pattern including at least 5 meals per day should be guaranteed. In addition, the quality and adequate composition of meals must be also promoted to children and, especially, their parents, families and caregivers.

Strength and limitations

Our findings are supported by a population-based cohort of children with more than 9 years of follow-up in a middle income country. Moreover, our longitudinal analysis shows strong association between meal frequency and BMI, taking into account the repeated-measurements and including changes of both variables over time. However, this study has limitations that should be highlighted. First, parents' reports were used to evaluate the number of meals per day based on the last day before the interview; recall bias may arise as a concern. In addition, parents may not be aware of eating frequency of children, especially at older ages. Moreover, although

For peer review only

questions assessed any meal or snack, portion (i.e. a large meal or a single bite) was not considered and, for instance, not evaluated as part of the analysis. Nevertheless, our results are congruent with previous studies (22-24). Second, other obesity markers, such as abdominal obesity, were not available for data analysis. Third, no all the potential confounders were available to adjust our regression models (e.g. physical activity or portion size); some residual confounding may be present. Finally, selection bias could be a concern as 5% of the richest districts were excluded during the sampling process, potentially affecting the broad generalizability of our results. Moreover, in countries undergoing nutrition transition such as Peru, changes in BMI are initially seen in the wealthiest individuals (25). Nonetheless, our estimates are informative for the general population though should be interpreted cautiously for families with high socio-economic status.

Conclusion

There is evidence of association between the number of meals per day consumed by a child and increasing BMI over time: those who ate less than 5 times per day had greater BMI and BMI-for-age z-score measurements compared to those eating ≥5 times per day. The promotion of a regular meal pattern including at least 5 meals per day should be guaranteed.

For peer review only

FUNDING STATEMENT

This research received no specific grant from any funding agency in the public, commercial or not-for-profit sectors. Rodrigo M Carrillo-Larco is supported by a Wellcome Trust International Training Fellowship (214185/Z/18/Z).

COMPETING INTEREST STATEMENT

The authors declare that no competing interests exist.

PATIENT AND PUBLIC INVOLVEMENT

Patients or the public were not involved in the design, or conduct, or reporting, or dissemination plans of our research.

AUTHORS' CONTRIBUTION

AB-O and RMC-L conceived the idea. RMC-L conducted the data cleaning and harmonization. AB-O conducted the statistical analysis. AB-O wrote the first version of the manuscript with input from RMC-L. Both authors approved the submitted version.

DATA SHARING STATEMENT

Data is available under request via the Young Lives webpage (https://www.younglives.org.uk/). Statistical code is available at: doi.org/10.6084/m9.figshare.11629092 (STATA syntax).

REFERENCES

1. NCD Risk Factor Collaboration (NCD-RisC). Worldwide trends in body-mass index, underweight, overweight, and obesity from 1975 to 2016: a pooled analysis of 2416 population-based measurement studies in 128.9 million children, adolescents, and adults. Lancet (London, England). 2017;390(10113):2627-42.
2. Rivera JA, de Cossio TG, Pedraza LS, Aburto TC, Sanchez TG, Martorell R. Childhood and adolescent overweight and obesity in Latin America: a systematic review. The lancet Diabetes & endocrinology. 2014;2(4):321-32.

For peer review only

3. Pajuelo-Ramirez J, Sanchez-Abanto J, Alvarez-Dongo D, Tarqui-Mamani C, Aguero-Zamora R. [Overweight, obesity and chronic mal nutrition in 6 to 9 year-old children in Peru, 2009-2010]. Revista peruana de medicina experimental y salud publica. 2013;30(4):583-9.

4. Romieu I, Dossus L, Barquera S, Blottiere HM, Franks PW, Gunter M, et al. Energy balance and obesity: what are the main drivers? Cancer causes & control : CCC. 2017;28(3):247-58.

5. Toschke AM, Kuchenhoff H, Koletzko B, von Kries R. Meal frequency and childhood obesity. Obesity research. 2005;13(11):1932-8.

6. Jaaskelainen A, Schwab U, Kolehmainen M, Pirkola J, Jarvelin MR, Laitinen J. Associations of meal frequency and breakfast with obesity and metabolic syndrome traits in adolescents of Northern Finland Birth Cohort 1986. Nutrition, metabolism, and cardiovascular diseases : NMCD. 2013;23(10):1002-9.

7. Koletzko B, Toschke AM. Meal patterns and frequencies: do they affect body weight in children and adolescents? Critical reviews in food science and nutrition. 2010;50(2):100-5.

8. Toschke AM, Thorsteinsdottir KH, von Kries R. Meal frequency, breakfast consumption and childhood obesity. International journal of pediatric obesity : IJPO : an official journal of the International Association for the Study of Obesity. 2009;4(4):242-8.

9. Lee SY, Ha SA, Seo JS, Sohn CM, Park HR, Kim KW. Eating habits and eating behaviors by family dinner frequency in the lower-grade elementary school students. Nutrition research and practice. 2014;8(6):679-87.

10. Mamun AA, Lawlor DA, O'Callaghan MJ, Williams GM, Najman JM. Positive maternal attitude to the family eating together decreases the risk of adolescent overweight. Obesity research. 2005;13(8):1422-30.

For peer review only

11. Dallacker M, Hertwig R, Mata J. The frequency of family meals and nutritional health in children: a meta-analysis. Obesity reviews : an official journal of the International Association for the Study of Obesity. 2018;19(5):638-53.
12. Serrano M, Torres R, Pérez CM, Palacios C. Social environment factors, diet quality, and body weight in 12-year-old children from four public schools in Puerto Rico. P R Health Sci J. 2014;33(2):80-7.
13. Ambikapathi R, Rothstein JD, Peñataro Yori P, Paredes Olortegui M, Lee G, Kosek MN, et al. Food purchase patterns indicative of household food access insecurity, children's dietary diversity and intake, and nutritional status using a newly developed and validated tool in the Peruvian Amazon. Food Secur. 2018;10(4):999-1011.
14. Vargas S, Penny ME. Measuring food insecurity and hunger in Peru: a qualitative and quantitative analysis of an adapted version of the USDA's Food Insecurity and Hunger Module. Public Health Nutr. 2010;13(10):1488-97.
15. Barnett I, Ariana P, Petrou S, Penny ME, Duc le T, Galab S, et al. Cohort profile: the Young Lives study. International journal of epidemiology. 2013;42(3):701-8.
16. Escobal J, Flores E. An Assessment of the Young Lives Sampling Approach in Peru. Technical Note 3. Oxford, United Kingdom: Young Lives, 2008.
17. Must A, Anderson SE. Body mass index in children and adolescents: considerations for population-based applications. International journal of obesity (2005). 2006;30(4):590-4.
18. World Health Organization. Obesity and overweight: WHO; 2018 [cited 2020 January 7]. Available from: https://www.who.int/news-room/fact-sheets/detail/obesity-and-overweight.

For peer review only

19. Valdes J, Rodriguez-Artalejo F, Aguilar L, Jaen-Casquero MB, Royo-Bordonada MA. Frequency of family meals and childhood overweight: a systematic review. Pediatric obesity. 2013;8(1):e1-e13.

20. Jenkins DJ, Khan A, Jenkins AL, Illingworth R, Pappu AS, Wolever TM, et al. Effect of nibbling versus gorging on cardiovascular risk factors: serum uric acid and blood lipids. Metabolism: clinical and experimental. 1995;44(4):549-55.

21. Ardeshirlarijani E, Namazi N, Jabbari M, Zeinali M, Gerami H, Jalili RB, et al. The link between breakfast skipping and overweigh/obesity in children and adolescents: a meta-analysis of observational studies. Journal of diabetes and metabolic disorders. 2019;18(2):657-64.

22. Gable S, Chang Y, Krull JL. Television watching and frequency of family meals are predictive of overweight onset and persistence in a national sample of school-aged children. Journal of the American Dietetic Association. 2007;107(1):53-61.

23. Laurson K, Eisenmann JC, Moore S. Lack of association between television viewing, soft drinks, physical activity and body mass index in children. Acta paediatrica (Oslo, Norway : 1992). 2008;97(6):795-800.

24. Taveras EM, Rifas-Shiman SL, Berkey CS, Rockett HR, Field AE, Frazier AL, et al. Family dinner and adolescent overweight. Obesity research. 2005;13(5):900-6.

25. Popkin BM, Adair LS, Ng SW. Global nutrition transition and the pandemic of obesity in developing countries. Nutrition reviews. 2012;70(1):3-21.

For peer review only

TABLES AND FIGURES

Figure 1: Flowchart of participants analyzed using the Young Lives younger cohort

For peer review only

Figure 2: Variation over time in (A) BMI and (B) BMI-for-age z-score according to meal frequency controlling for potential confounders

(A) (B)

For peer review only

Table 1: Characteristics of the study population according to meal frequency at baseline

	Meal frequency at baseline (times per day)					
	< 4 (n = 217)	4 (n = 402)	5 (n = 851)	6 (n = 409)	7 (n = 77)	p-value*
**Sex, n (%)**						0.52
Female	102 (47.0%)	201 (50.0%)	438 (51.5%)	191 (46.7%)	39 (50.7%)	
Male	115 (53.0%)	201 (50.0%)	413 (48.5%)	218 (53.3%)	38 (49.4%)	
**Age, mean (SD)**						< 0.001
In years	4.2 (0.2)	4.3 (0.3)	4.4 (0.3)	4.3 (0.3)	4.3 (0.2)	
**Maternal education, n (%)**						< 0.001
< 7 years	161 (74.5%)	219 (54.9%)	312 (37.1%)	150 (36.7%)	28 (36.8%)	
7 – 11 years	47 (21.8%)	139 (34.8%)	340 (40.4%)	158 (38.6%)	28 (36.8%)	
12+ years	8 (3.7%)	41 (10.3%)	189 (22.5%)	101 (24.7%)	20 (26.3%)	
**Paternal education, n (%)**						< 0.001
< 7 years	116 (56.0%)	149 (38.3%)	248 (30.0%)	112 (28.3%)	24 (32.0%)	
7 – 11 years	80 (38.7%)	181 (46.5%)	369 (44.6%)	185 (46.7%)	27 (36.0%)	
12+ years	11 (5.3%)	59 (15.2%)	211 (25.4%)	99 (25.0%)	24 (32.0%)	
**Socioeconomic status, n (%)**						< 0.001
Low	144 (66.3%)	167 (41.6%)	219 (25.7%)	100 (24.5%)	24 (31.2%)	
Middle	57 (26.3%)	148 (36.8%)	283 (33.3%)	140 (34.2%)	25 (32.5%)	
High	16 (7.4%)	87 (21.6%)	349 (41.0%)	169 (41.3%)	28 (36.4%)	
**Setting type, n (%)**						< 0.001
Urban	53 (24.4%)	195 (48.5%)	544 (63.9%)	248 (60.6%)	42 (54.6%)	
Rural	164 (75.6%)	207 (51.5%)	307 (36.1%)	161 (39.4%)	35 (45.4%)	
**Maternal BMI, mean (SD)**						0.02
In Kg/m^2	25.3 (3.3)	25.9 (4.1)	26.4 (4.5)	26.1 (3.8)	25.8 (3.7)	
**Maternal BMI, n (%)**						0.10

For peer review only

Normal	108 (52.2%)	175 (46.1%)	343 (42.6%)	171 (43.4%)	33 (46.5%)
Overweight	84 (40.6%)	148 (38.9%)	333 (41.4%)	162 (41.1%)	29 (40.8%)
Obese	15 (7.2%)	57 (15.0%)	129 (16.0%)	61 (15.5%)	9 (12.7%)

SD = standard deviation; BMI = Body mass index
* Comparisons were conducted using Chi-squared tests for categorical variables, and analysis of variance for numerical ones.

For peer review only

Table 2: BMI and BMI z-score by meal frequency at baseline: Mean and standard deviation (SD) during follow-up

	Mean and standard deviation of BMI and BMI z-score			
	**Baseline (n = 1948)**	**First follow-up (n = 1904)**	**Second follow-up (n = 1850)**	**Third follow-up (n = 1810)**
**Age**				
Mean age (SD)	4.3 (0.3)	6.9 (0.7)	10.9 (0.1)	13.9 (0.1)
**Meal frequency**	**BMI (in Kg/m²): mean (SD)**			
< 4 times per day	16.4 (1.9)	16.4 (1.7)	18.5 (2.5)	21.1 (2.7)
4 times per day	16.4 (1.5)	16.8 (2.1)	19.4 (3.0)	21.7 (3.3)
5 times per day	16.5 (1.9)	17.0 (2.4)	19.9 (3.4)	21.8 (3.4)
6 times per day	16.3 (1.6)	16.9 (2.4)	19.8 (3.3)	21.6 (3.4)
7 times per day	16.5 (2.4)	16.8 (2.4)	19.4 (3.3)	21.0 (2.9)
**Meal frequency**	**BMI z-score: mean (SD)**			
< 4 times per day	0.67 (1.06)	0.29 (0.92)	0.18 (0.92)	0.29 (0.81)
4 times per day	0.68 (0.92)	0.49 (1.02)	0.50 (1.02)	0.44 (0.93)
5 times per day	0.71 (1.09)	0.59 (1.08)	0.64 (1.11)	0.44 (1.01)
6 times per day	0.65 (0.98)	0.55 (1.13)	0.61 (1.08)	0.41 (0.99)
7 times per day	0.72 (1.33)	0.42 (1.14)	0.45 (1.15)	0.26 (0.90)

SD = Standard deviation; BMI = body mass index

For peer review only

Table 3: Meal frequency and changes over time of body mass index: crude and adjusted linear mixed models

	**Crude model**<br>**Coefficient (95% CI)**	**Adjusted model***<br>**Coefficient (95% CI)**
**Meal frequency**	**Change in BMI (Kg/m^2)**	
< 4 times per day	**1.18 (0.73; 1.64)**	**0.39 (0.17; 0.62)**
4 times per day	**0.71 (0.43; 0.98)**	**0.16 (0.02; 0.30)**
5 times per day	1 (Reference)	1 (Reference)
6 times per day	**-0.54 (-0.68; -0.40)**	-0.03 (-0.15; 0.09)
7 times per day	**-0.73 (-1.26; -0.21)**	0.08 (-0.33; 0.50)
**Meal frequency**	**Change in BMI z-score**	
< 4 times per day	0.01 (-0.08; 0.10)	**0.07 (0.01; 0.13)**
4 times per day	0.01 (-0.03; 0.06)	**0.05 (0.01; 0.09)**
5 times per day	1 (Reference)	1 (Reference)
6 times per day	0.01 (-0.03; 0.06)	-0.01 (-0.05; 0.04)
7 times per day	0.06 (-0.10; 0.21)	0.04 (-0.13; 0.20)

95% CI: 95% confidence intervals; BMI = body mass index
* Adjusted for sex, age, maternal education, paternal education, wealth index, setting type, and maternal BMI (in categories). Estimates in bold are statistical significant.

For peer review only

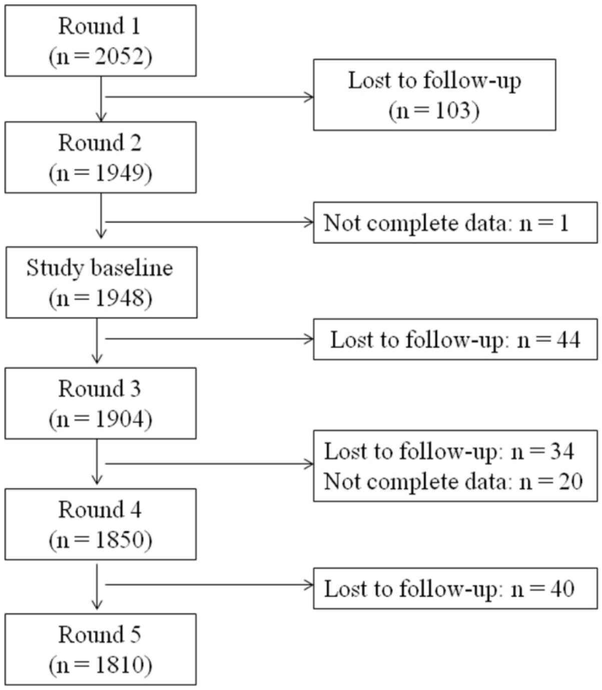

Figure 1: Flowchart of participants

150x112mm (300 x 300 DPI)

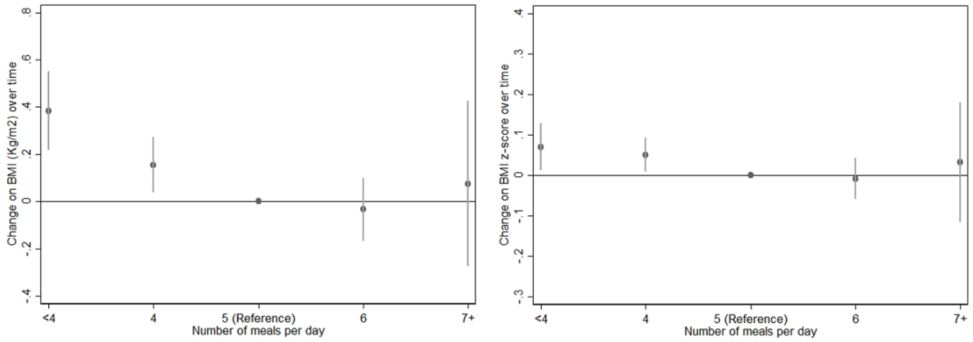

Figure 2: Changes of BMI and BMI-for-age z-score over time according to meal frequency

250x92mm (300 x 300 DPI)

STROBE 2007 (v4) Statement—Checklist of items that should be included in reports of *cohort studies*

Section/Topic	Item #	Recommendation	Reported on page #
**Title and abstract**	1	(*a*) Indicate the study's design with a commonly used term in the title or the abstract	1
		(*b*) Provide in the abstract an informative and balanced summary of what was done and what was found	3
**Introduction**			
Background/rationale	2	Explain the scientific background and rationale for the investigation being reported	6
Objectives	3	State specific objectives, including any prespecified hypotheses	6
**Methods**			
Study design	4	Present key elements of study design early in the paper	7
Setting	5	Describe the setting, locations, and relevant dates, including periods of recruitment, exposure, follow-up, and data collection	7
Participants	6	(*a*) Give the eligibility criteria, and the sources and methods of selection of participants. Describe methods of follow-up	7
		(*b*) For matched studies, give matching criteria and number of exposed and unexposed	
Variables	7	Clearly define all outcomes, exposures, predictors, potential confounders, and effect modifiers. Give diagnostic criteria, if applicable	8
Data sources/ measurement	8*	For each variable of interest, give sources of data and details of methods of assessment (measurement). Describe comparability of assessment methods if there is more than one group	8
Bias	9	Describe any efforts to address potential sources of bias	9
Study size	10	Explain how the study size was arrived at	9
Quantitative variables	11	Explain how quantitative variables were handled in the analyses. If applicable, describe which groupings were chosen and why	9
Statistical methods	12	(*a*) Describe all statistical methods, including those used to control for confounding	9
		(*b*) Describe any methods used to examine subgroups and interactions	9
		(*c*) Explain how missing data were addressed	NA
		(*d*) If applicable, explain how loss to follow-up was addressed	NA
		(*e*) Describe any sensitivity analyses	NA
**Results**			

For peer review only

Participants	13*	(a) Report numbers of individuals at each stage of study—eg numbers potentially eligible, examined for eligibility, confirmed eligible, included in the study, completing follow-up, and analysed	10
		(b) Give reasons for non-participation at each stage	10
		(c) Consider use of a flow diagram	Figure 1
Descriptive data	14*	(a) Give characteristics of study participants (eg demographic, clinical, social) and information on exposures and potential confounders	10
		(b) Indicate number of participants with missing data for each variable of interest	Figure 1
		(c) Summarise follow-up time (eg, average and total amount)	10
Outcome data	15*	Report numbers of outcome events or summary measures over time	10
Main results	16	(*a*) Give unadjusted estimates and, if applicable, confounder-adjusted estimates and their precision (eg, 95% confidence interval). Make clear which confounders were adjusted for and why they were included	10-11
		(*b*) Report category boundaries when continuous variables were categorized	10-11
		(*c*) If relevant, consider translating estimates of relative risk into absolute risk for a meaningful time period	10-11
Other analyses	17	Report other analyses done—eg analyses of subgroups and interactions, and sensitivity analyses	10-11
**Discussion**			
Key results	18	Summarise key results with reference to study objectives	11
**Limitations**			
Interpretation	20	Give a cautious overall interpretation of results considering objectives, limitations, multiplicity of analyses, results from similar studies, and other relevant evidence	11-12
Generalisability	21	Discuss the generalisability (external validity) of the study results	11-12
**Other information**			
Funding	22	Give the source of funding and the role of the funders for the present study and, if applicable, for the original study on which the present article is based	13

*Give information separately for cases and controls in case-control studies and, if applicable, for exposed and unexposed groups in cohort and cross-sectional studies.

Note: An Explanation and Elaboration article discusses each checklist item and gives methodological background and published examples of transparent reporting. The STROBE checklist is best used in conjunction with this article (freely available on the Web sites of PLoS Medicine at http://www.plosmedicine.org/, Annals of Internal Medicine at http://www.annals.org/, and Epidemiology at http://www.epidem.com/). Information on the STROBE Initiative is available at www.strobe-statement.org.

For peer review only

BMJ Open

Longitudinal association between food frequency and changes in body mass index: a prospective cohort study

Journal:	*BMJ Open*
Manuscript ID	bmjopen-2020-037057.R2
Article Type:	Original research
Date Submitted by the Author:	21-Apr-2020
Complete List of Authors:	Bernabe-Ortiz, Antonio; Universidad Peruana Cayetano Heredia, CRONICAS Centre of Excellence in Chronic Diseases<br>Carrillo-Larco, Rodrigo; Universidad Peruana Cayetano Heredia, CRONICAS Center of Excellence; CRONICAS Center of Excellence in Chronic Diseases
**Primary Subject Heading**:	Nutrition and metabolism
Secondary Subject Heading:	Epidemiology, Public health
Keywords:	EPIDEMIOLOGY, NUTRITION & DIETETICS, PUBLIC HEALTH

SCHOLARONE™
Manuscripts

I, the Submitting Author has the right to grant and does grant on behalf of all authors of the Work (as defined in the below author licence), an exclusive licence and/or a non-exclusive licence for contributions from authors who are: i) UK Crown employees; ii) where BMJ has agreed a CC-BY licence shall apply, and/or iii) in accordance with the terms applicable for US Federal Government officers or employees acting as part of their official duties; on a worldwide, perpetual, irrevocable, royalty-free basis to BMJ Publishing Group Ltd ("BMJ") its licensees and where the relevant Journal is co-owned by BMJ to the co-owners of the Journal, to publish the Work in this journal and any other BMJ products and to exploit all rights, as set out in our licence.

The Submitting Author accepts and understands that any supply made under these terms is made by BMJ to the Submitting Author unless you are acting as an employee on behalf of your employer or a postgraduate student of an affiliated institution which is paying any applicable article publishing charge ("APC") for Open Access articles. Where the Submitting Author wishes to make the Work available on an Open Access basis (and intends to pay the relevant APC), the terms of reuse of such Open Access shall be governed by a Creative Commons licence – details of these licences and which Creative Commons licence will apply to this Work are set out in our licence referred to above.

Other than as permitted in any relevant BMJ Author's Self Archiving Policies, I confirm this Work has not been accepted for publication elsewhere, is not being considered for publication elsewhere and does not duplicate material already published. I confirm all authors consent to publication of this Work and authorise the granting of this licence.

For peer review only

Longitudinal association between food frequency and changes in body mass index: a prospective cohort study

Antonio Bernabe-Ortiz, MD, MPH, PhD[1,2]; Rodrigo M. Carrillo-Larco, MD[1,3,4]

1. CRONICAS Center of Excellence in Chronic Diseases, Universidad Peruana Cayetano Heredia, Lima, Peru
2. Universidad Cientifica del Sur, Lima, Perú
3. Department of Epidemiology and Biostatistics, School of Public Health, Imperial College London, London, UK
4. Centro de Estudios de Población, Universidad Católica los Ángeles de Chimbote (ULADECH-Católica), Chimbote, Peru

Short title:

Meal frequency and body mass index

E-mails:

Antonio Bernabe-Ortiz: Antonio.Bernabe@upch.pe

Rodrigo M. Carrillo-Larco: Rodrigo.Carrillo@upch.pe

Corresponding autor:

Antonio Bernabe-Ortiz, MD, MPH, PhD

CRONICAS Center of Excellence in Chronic Diseases, Universidad Peruana Cayetano Heredia

For peer review only

Av. Armendariz 445, Miraflores, Lima, Peru

E-mail: Antonio.Bernabe@upch.pe

Phone: 51-1-241-6978

Word count:

Abstract: 229 words

Manuscript (from Introduction to References): 3000 words (total: 4318 words)

For peer review only

ABSTRACT

Objectives: Analyzing data of the Young Lives Study in Peru, we aimed at assessing the association between daily food frequency and BMI changes between 2006 and 2016.

Design: Secondary analysis of a prospective ongoing cohort study.

Setting: 20 sentinel sites in Peru

Participants: Children enrolled in the younger cohort of the Young Lives Study. We used information from the second (2006-07), third (2009-10), fourth (2013-14), and fifth (2016-17) rounds of the younger cohort in Peru.

Primary and secondary outcomes: Body mass index (BMI) as well as BMI-for-age z-score, both as numerical variables.

Results: Data from 1948 children, mean age 4.3 (SD: 0.3) years and 966 (49.6%) females were included at baseline. In multivariable model, lower food consumption frequency was associated with increased BMI and BMI-for-age z-scores: children reporting <4 times of food consumption per day had a greater increase in BMI ($\beta = 0.39$; 95%CI: 0.17; 0.62) and BMI-for-age z-score ($\beta = 0.07$; 95%CI: 0.01; 0.13) compared to those reporting 5 per day. Results were consistent for those reporting exactly eating 4 times per day (β for BMI = 0.16; 95%CI: 0.02; 0.30, and β for BMI-for-age z-score = 0.05; 95%CI: 0.01; 0.09).

Conclusions: Children who eat <5 times per day, gain more BMI compared to those who eat ≥5 times. Parents should receive information to secure adequate nutrition for their children, both in terms of quality and quantity.

Keywords: Body mass index, meal frequency, longitudinal study, children, adolescent.

For peer review only

STRENGHTS AND LIMITATIONS OF THIS STUDY

- Results are supported by a population-based cohort of children with more than nine years of follow-up in a middle income country.
- Our analysis took into account the repeated-measure nature of the data including changes of both outcome and exposure over time.
- Recall bias can be an issue as mother's reports were used to evaluate the number of times of food consumption per day.

For peer review only

INTRODUCTION

Current trends in children's and adolescent's body mass index (BMI) show a sustained and fast growth in different low- and middle-income countries (LMIC) (1). Moreover, the swift transition from underweight to overweight and obesity has also been reported in specific countries in Latin America, where the median of the prevalence distribution of excess bodyweight among school-aged children has been estimated in 29.8%, while this number was 17.8% among adolescents (2). Similar figures have been found in Peru among children between 6 and 9 years old (3).

Changes in BMI are explained by a misbalance between food intake and energy expenditure (4); therefore, some diet characteristics, such as diet quality and excess energy intake, may affect these changes. In that sense, food frequency has been described as a potential risk factor for BMI increase and, for instance, overweight and obesity (5).

Some studies have reported that regular (i.e. more frequent) food frequency is associated with lower risk of overweight and obesity (6-8), whereas other studies have not reported such association (9, 10). Moreover, a relatively recent systematic review and meta-analysis of 57 observational studies reported a significant relationship between frequent meals and better nutrition outcomes, including lower BMI (11). Nevertheless, this meta-analysis included only reports from high-income countries, showed correlations because most of the summarized studies were cross-sectional (only eight studies were longitudinal), and only one study was from Latin America (Puerto Rico) (12). Moreover, among those longitudinal studies, the variation of food frequency and BMI over time was not taken into account. Therefore, whether the association between food frequency and BMI holds in Latin America, is unknown; though there could be differences because of the nutritional profiles and access to food patterns of these populations. For example, food insecurity is high in Peru, especially in rural and poor areas (13),

For peer review only

and households with an insecure profile usually have a poor diet quality and consume more carbohydrates (14). Thus, this study aimed at assessing the association between food frequency and BMI changes over time among children in Peru. We hypothesized that children eating less frequently will have a greater gain of BMI over time.

METHODS

Study design

This is a secondary analysis utilizing information of the Young Lives Study, a longitudinal ongoing prospective cohort conducted in four developing countries (Ethiopia, India, Peru and Vietnam) (15). The cohort started in 2002 and currently has five assessment rounds, i.e. a baseline and four follow-up evaluations.

Study participants

The Young Lives Study in Peru is comprised by two different cohorts: the younger cohort that initially enrolled 2052 children aged between 6 and 18 months, and the older cohort that included 1000 children between 7 and 8 years (15). For this work, we utilized information from the second (2006-07), third (2009-10), fourth (2013-14), and fifth (2016-17) rounds of the younger cohort in Peru as information about food frequency was available in that cohort. As a result, information of the second round was used as the baseline of the cohort, and information from the third, fourth and fifth rounds were included as follow-up assessments. Records with incomplete information in the variables of interest (food frequency and body mass index) were excluded from the analysis.

Sampling

For peer review only

A sentinel site sampling strategy was used as previously published (16). Briefly, a multistage, cluster stratified, random sampling technique of sentinel sites was conducted. The initial sample frame was carried out at the district level, selecting 20 sentinel sites from a total of 1818 districts available. To oversampling poor areas, the top 5% richest districts were not included in the sampling process.

Maps of census tracts, comprising block of houses or set of houses, were used to randomly select one census tract in each district utilizing a random number table. All households in any given block or set of houses were visited to identify one household with at least one child meeting inclusion criteria. Different block or set of houses were approached until the total eligible households were completed. Three different teams, including fieldworkers, data-entry staff and supervisors were responsible for data collection and quality of six or seven sentinel sites.

Study variables

Two were the outcomes of interest for this study: body mass index (BMI) evaluated as numerical continuous variable and BMI-for-age z-score. Both height and weight were measured in each children assessment and used to estimate BMI as usual. BMI-for-age z-score was based on the World Health Organization reference (17). We used these outcomes because we aimed at assessing changes over time using the information available from all the four study rounds. In addition, both definitions (i.e. BMI and BMI-for-age z-score) were used as BMI alone is not the best indicator of excess of weight among children under six years of age (17, 18).

The exposure of interest was food frequency as reported by the parents based on seven questions related to the consumption of any possible meal or snack during the previous 24 hours before the interview. The question was asked as follow: "During the previous 24-hour period, did the child consume: any food before a morning meal (breakfast)? Any morning meal (breakfast)? Any food

For peer review only

between morning and midday meals? Any midday meal? Any food between midday and evening meals? Any evening meal? Any food after the evening meal? A simple addition of yes/no responses to these seven questions was used to estimate the total food frequency the child ate in the last 24 hours. For analysis purposes, the variable was categorized as <4, 4, 5, 6, and 7 times per day (5), and the middle category (5 times per day) was used as the reference category. This process was conducted for each assessment included in the analysis (i.e. baseline and three follow-up evaluations).

Other variables were also included as potential confounders in regression models as evaluated at baseline: sex (boy or girl), age (in years), socioeconomic status assessed using a wealth index created based on household assets and split in tertiles (low, middle and high), setting type (urban or rural), maternal and paternal education based on years of education (<7 years, 7-11 years, and ≥12 years), and maternal BMI as continuous variable, but also split according to traditional thresholds (normal [<25 kg/m^2], overweight [25-29 kg/m^2] and obese [≥30 kg/m^2]) (18).

Data analysis

STATA 13 for Windows (StataCorp, College Station, TX, US) was used for statistical analysis. Initially, the description of the study population was tabulated according to food frequency using mean and standard deviation (SD) for numerical variables and proportions for categorical ones.

Chi-squared test or analysis of variance was used for comparison between variables accordingly.

Means and standard deviation (SD) of BMI and BMI-for-age z-score for baseline and each follow-up were calculated according to food frequency at baseline (i.e. without taking into account correlation between measurements or any other variables). Finally, crude and adjusted linear mixed models with random intercepts were used to determine the association of interest (i.e. association between BMI or BMI-for-age z-score and food frequency, accounting for the

For peer review only

repeated-measure nature of the study and including changes of both variables over time). Three levels were fitted in the regression models (children assessment as level 1, subject as level 2, and sentinel site as level 3). BMI changes over time were presented as coefficients and their respective 95% confidence intervals (95% CI) controlling for different confounders.

Ethics

The Young Lives study was originally approved by the Ethics Committee Social Science Division, University of Oxford, UK, in 2002. In Peru, the approval was granted by the Research Ethics Committee of the Instituto de Investigacion Nutricional in Lima. Data is freely available under request and information is de-identified.

RESULTS

Baseline characteristics of the study population

A total of 2052 children were originally recruited in the younger cohort in Peru. Of them, 103 (5.0%) were lost to follow-up and 1 (0.1%) was excluded because of missing data (Figure 1). Thus, 1948 individuals were included in the baseline analysis, with a mean age of 4.3 (SD: 0.3) years and 966 (49.6%) were females. At baseline, food consumption frequency was on average 4.9 (SD: 1.0, range: 2 – 7) times per day and 847 (43.5%) reported eating 5 times per day, whereas 215 (11.0%) ate <4 times per day and 77 (4.0%) ate ≥ 7 times per day. The characteristics of the study population according to food frequency at baseline are shown in Table 1. Of note, children age, maternal and paternal education, socioeconomic status, setting type, and maternal BMI were positively associated with food frequency.

Food frequency and body mass index during follow-up

For peer review only

Figure 1 shows in detail the number of participants in each of the follow-up assessments. On average, children were followed-up for a total of 9.6 (SD: 0.3) years. The proportion of children that reported eating ≥5 times per day dropped from 68.4% at baseline (4.3 ± 0.3 years of age) to 49.8% at the end of the follow-up (13.9 ± 0.1 years of age).

Table 2 shows a description of the mean BMI during each study follow-up rounds without taking into account intra-subject correlation. All the food frequency groups showed an increase of BMI and BMI-for-age z-score, and this increase seemed to be higher in the group reporting a food frequency of 5 times per day.

BMI and BMI-for-age z-score change over time

Results of the crude and adjusted regression models are shown in Table 3. According to multivariable model, our results show that lower food frequency consumption was associated with increased BMI and BMI-for-age z-scores. Children reporting <4 meals per day had a greater increase in BMI (β = 0.39; 95%CI: 0.17; 0.62) and BMI-for-age z-score (β = 0.07; 95%CI: 0.01; 0.13) compared to those reporting eating 5 times per day. These results were similar for those reporting eating exactly 4 times per day (β for BMI = 0.16; 95%CI: 0.02; 0.30, and β for BMI-for-age z-score = 0.05; 95%CI: 0.01; 0.09); however, changes among those eating 6 or 7 times per day did not show association (See Figure 2).

DISCUSSION

Main findings

Our study shows evidence of an association between daily food frequency and children's BMI changes over time. Although the magnitude of the association was small, less frequent food consumption was associated with greater BMI gain over time: those eating less than 5 times per

For peer review only

day had greater BMI and BMI-for-age z-score measurements, whereas those eating ≥5 times per day did not have an increase in these outcomes. In addition, almost two thirds of the study population reported eating ≥5 times per day at baseline (i.e. when the child aged between 4-5 years), but this number reduced to less than half of participants when children were between 14 and 15 years.

Comparison with previous studies

Two systematic reviews have approximated to the association proposed in this paper. The first one reported inconsistent and weak evidence of an inverse association between food frequency and risk of childhood overweight (19). This review used 15 reports and only 4 were longitudinal studies. The second systematic review, conducted in 2018, found a significant relationship between frequent food consumption and better nutritional health using 57 studies, both in younger and older children, across countries and socioeconomic groups (11). However, the latter review only reported weighted mean correlations and no risk of overnutrition or changes of BMI over time, and many of the summarized reports studied convenience samples. Our work agrees with the current literature and adds more evidence from new populations (children in Latin America as limited information is available) and followed a strong observational design (prospective cohort).

Many of the studies looking for the association of interest have been cross-sectional, and have used outcomes based on overweight or obesity. Toschke et al reported a decrease in the prevalence of obesity by number of daily meals in two different studies (5, 8); whereas in a cross-sectional assessment of a Finnish cohort study of adolescents they reported that adolescents who ate five meals/day were at lower risk for general and abdominal obesity as well as hypertriglyceridemia (6). Thus, our study expands on previous findings by using repeated

For peer review only

measurements following appropriate longitudinal analysis to verify the association of interest in a population-based cohort study of children transitioning to adolescents.

Of note, our findings support the fact that the number of food consumed per day seems to decrease from childhood to adolescence. Therefore, children may lose the beneficial effects of eating more often throughout the day, thus increasing the probability of having overnutrition during adolescence.

Public health relevance

Our data suggest that a higher number of daily food consumption may reduce body mass index over time, reducing potentially the risk of overweight and obesity in children and adolescents. Effects of food frequency on endocrine responses and regulation might explain our findings. Increased food frequency may attenuate postprandial metabolic and endocrine responses to nutrient intake, with the subsequent reduction in insulin secretion (7, 20).

Since there is more evidence that skipping meals, especially breakfast, is associated with increasing risk of overnutrition (i.e. overweight and obesity) among children (21), the promotion of a regular food pattern including at least 5 meals per day should be guaranteed. In addition, the quality and adequate composition of meals must be also promoted to children and, especially, their parents, families and caregivers.

Strength and limitations

Our findings are supported by a population-based cohort of children with more than nine years of follow-up in a middle income country. Moreover, our longitudinal analysis shows strong association between food frequency and BMI, taking into account the repeated-measurements and including changes of both variables over time. However, this study has limitations that should be highlighted. First, parents' reports were used to evaluate the food frequency

For peer review only

consumption per day based on the last day before the interview; recall bias may arise as a concern. In addition, parents may not be aware of eating frequency of children, especially at older ages. Moreover, although questions assessed any meal or snack, portion (i.e. a large meal or a single bite) was not considered and, for instance, not evaluated as part of the analysis. Nevertheless, our results are congruent with previous studies (22-24). Second, other obesity markers, such as abdominal obesity, were not available for data analysis. Third, no all the potential confounders were available to adjust our regression models (e.g. physical activity or portion size); some residual confounding may be present. Finally, selection bias could be a concern as 5% of the richest districts were excluded during the sampling process, potentially affecting the broad generalizability of our results. Moreover, in countries undergoing nutrition transition such as Peru, changes in BMI are initially seen in the wealthiest individuals (25). Nonetheless, our estimates are informative for the general population though should be interpreted cautiously for families with high socio-economic status.

Conclusion

There is evidence of association between the number of foods consumed per day consumed by a child and increasing BMI over time: those who ate less than 5 times per day had greater BMI and BMI-for-age z-score measurements compared to those eating ≥5 times per day. The promotion of a regular food consumption pattern including at least 5 per day should be guaranteed.

FUNDING STATEMENT

This research received no specific grant from any funding agency in the public, commercial or not-for-profit sectors. Rodrigo M Carrillo-Larco is supported by a Wellcome Trust International Training Fellowship (214185/Z/18/Z).

For peer review only

COMPETING INTEREST STATEMENT

The authors declare that no competing interests exist.

PATIENT AND PUBLIC INVOLVEMENT

Patients or the public were not involved in the design, or conduct, or reporting, or dissemination plans of our research.

AUTHORS' CONTRIBUTION

AB-O and RMC-L conceived the idea. RMC-L conducted the data cleaning and harmonization. AB-O conducted the statistical analysis. AB-O wrote the first version of the manuscript with input from RMC-L. Both authors approved the submitted version.

DATA SHARING STATEMENT

Data is available under request via the Young Lives webpage (https://www.younglives.org.uk/). Statistical code is available at: doi.org/10.6084/m9.figshare.11629092 (STATA syntax).

REFERENCES

1. NCD Risk Factor Collaboration (NCD-RisC). Worldwide trends in body-mass index, underweight, overweight, and obesity from 1975 to 2016: a pooled analysis of 2416 population-based measurement studies in 128.9 million children, adolescents, and adults. Lancet (London, England). 2017;390(10113):2627-42.

For peer review only

2. Rivera JA, de Cossio TG, Pedraza LS, Aburto TC, Sanchez TG, Martorell R. Childhood and adolescent overweight and obesity in Latin America: a systematic review. The lancet Diabetes & endocrinology. 2014;2(4):321-32.
3. Pajuelo-Ramirez J, Sanchez-Abanto J, Alvarez-Dongo D, Tarqui-Mamani C, Aguero-Zamora R. [Overweight, obesity and chronic mal nutrition in 6 to 9 year-old children in Peru, 2009-2010]. Revista peruana de medicina experimental y salud publica. 2013;30(4):583-9.
4. Romieu I, Dossus L, Barquera S, Blottiere HM, Franks PW, Gunter M, et al. Energy balance and obesity: what are the main drivers? Cancer causes & control : CCC. 2017;28(3):247-58.
5. Toschke AM, Kuchenhoff H, Koletzko B, von Kries R. Meal frequency and childhood obesity. Obesity research. 2005;13(11):1932-8.
6. Jaaskelainen A, Schwab U, Kolehmainen M, Pirkola J, Jarvelin MR, Laitinen J. Associations of meal frequency and breakfast with obesity and metabolic syndrome traits in adolescents of Northern Finland Birth Cohort 1986. Nutrition, metabolism, and cardiovascular diseases : NMCD. 2013;23(10):1002-9.
7. Koletzko B, Toschke AM. Meal patterns and frequencies: do they affect body weight in children and adolescents? Critical reviews in food science and nutrition. 2010;50(2):100-5.
8. Toschke AM, Thorsteinsdottir KH, von Kries R. Meal frequency, breakfast consumption and childhood obesity. International journal of pediatric obesity : IJPO : an official journal of the International Association for the Study of Obesity. 2009;4(4):242-8.
9. Lee SY, Ha SA, Seo JS, Sohn CM, Park HR, Kim KW. Eating habits and eating behaviors by family dinner frequency in the lower-grade elementary school students. Nutrition research and practice. 2014;8(6):679-87.

For peer review only

10. Mamun AA, Lawlor DA, O'Callaghan MJ, Williams GM, Najman JM. Positive maternal attitude to the family eating together decreases the risk of adolescent overweight. Obesity research. 2005;13(8):1422-30.

11. Dallacker M, Hertwig R, Mata J. The frequency of family meals and nutritional health in children: a meta-analysis. Obesity reviews : an official journal of the International Association for the Study of Obesity. 2018;19(5):638-53.

12. Serrano M, Torres R, Pérez CM, Palacios C. Social environment factors, diet quality, and body weight in 12-year-old children from four public schools in Puerto Rico. P R Health Sci J. 2014;33(2):80-7.

13. Ambikapathi R, Rothstein JD, Peñataro Yori P, Paredes Olortegui M, Lee G, Kosek MN, et al. Food purchase patterns indicative of household food access insecurity, children's dietary diversity and intake, and nutritional status using a newly developed and validated tool in the Peruvian Amazon. Food Secur. 2018;10(4):999-1011.

14. Vargas S, Penny ME. Measuring food insecurity and hunger in Peru: a qualitative and quantitative analysis of an adapted version of the USDA's Food Insecurity and Hunger Module. Public Health Nutr. 2010;13(10):1488-97.

15. Barnett I, Ariana P, Petrou S, Penny ME, Duc le T, Galab S, et al. Cohort profile: the Young Lives study. International journal of epidemiology. 2013;42(3):701-8.

16. Escobal J, Flores E. An Assessment of the Young Lives Sampling Approach in Peru. Technical Note 3. Oxford, United Kingdom: Young Lives, 2008.

17. Must A, Anderson SE. Body mass index in children and adolescents: considerations for population-based applications. International journal of obesity (2005). 2006;30(4):590-4.

For peer review only

18. World Health Organization. Obesity and overweight: WHO; 2018 [cited 2020 January 7]. Available from: https://www.who.int/news-room/fact-sheets/detail/obesity-and-overweight.

19. Valdes J, Rodriguez-Artalejo F, Aguilar L, Jaen-Casquero MB, Royo-Bordonada MA. Frequency of family meals and childhood overweight: a systematic review. Pediatric obesity. 2013;8(1):e1-e13.

20. Jenkins DJ, Khan A, Jenkins AL, Illingworth R, Pappu AS, Wolever TM, et al. Effect of nibbling versus gorging on cardiovascular risk factors: serum uric acid and blood lipids. Metabolism: clinical and experimental. 1995;44(4):549-55.

21. Ardeshirlarijani E, Namazi N, Jabbari M, Zeinali M, Gerami H, Jalili RB, et al. The link between breakfast skipping and overweigh/obesity in children and adolescents: a meta-analysis of observational studies. Journal of diabetes and metabolic disorders. 2019;18(2):657-64.

22. Gable S, Chang Y, Krull JL. Television watching and frequency of family meals are predictive of overweight onset and persistence in a national sample of school-aged children. Journal of the American Dietetic Association. 2007;107(1):53-61.

23. Laurson K, Eisenmann JC, Moore S. Lack of association between television viewing, soft drinks, physical activity and body mass index in children. Acta paediatrica (Oslo, Norway : 1992). 2008;97(6):795-800.

24. Taveras EM, Rifas-Shiman SL, Berkey CS, Rockett HR, Field AE, Frazier AL, et al. Family dinner and adolescent overweight. Obesity research. 2005;13(5):900-6.

25. Popkin BM, Adair LS, Ng SW. Global nutrition transition and the pandemic of obesity in developing countries. Nutrition reviews. 2012;70(1):3-21.

For peer review only

TABLES AND FIGURES

Figure 1: Flowchart of participants analyzed using the Young Lives younger cohort

For peer review only

Figure 2: Variation over time in (A) BMI and (B) BMI-for-age z-score according to food frequency controlling for potential confounders

(A)

(B)

For peer review only

Table 1: Characteristics of the study population according to food frequency at baseline

	Food frequency at baseline (times per day)					
	< 4 (n = 217)	4 (n = 402)	5 (n = 851)	6 (n = 409)	7 (n = 77)	p-value*
**Sex, n (%)**						0.52
Female	102 (47.0%)	201 (50.0%)	438 (51.5%)	191 (46.7%)	39 (50.7%)	
Male	115 (53.0%)	201 (50.0%)	413 (48.5%)	218 (53.3%)	38 (49.4%)	
**Age, mean (SD)**						< 0.001
In years	4.2 (0.2)	4.3 (0.3)	4.4 (0.3)	4.3 (0.3)	4.3 (0.2)	
**Maternal education, n (%)**						< 0.001
< 7 years	161 (74.5%)	219 (54.9%)	312 (37.1%)	150 (36.7%)	28 (36.8%)	
7 – 11 years	47 (21.8%)	139 (34.8%)	340 (40.4%)	158 (38.6%)	28 (36.8%)	
12+ years	8 (3.7%)	41 (10.3%)	189 (22.5%)	101 (24.7%)	20 (26.3%)	
**Paternal education, n (%)**						< 0.001
< 7 years	116 (56.0%)	149 (38.3%)	248 (30.0%)	112 (28.3%)	24 (32.0%)	
7 – 11 years	80 (38.7%)	181 (46.5%)	369 (44.6%)	185 (46.7%)	27 (36.0%)	
12+ years	11 (5.3%)	59 (15.2%)	211 (25.4%)	99 (25.0%)	24 (32.0%)	
**Socioeconomic status, n (%)**						< 0.001
Low	144 (66.3%)	167 (41.6%)	219 (25.7%)	100 (24.5%)	24 (31.2%)	
Middle	57 (26.3%)	148 (36.8%)	283 (33.3%)	140 (34.2%)	25 (32.5%)	
High	16 (7.4%)	87 (21.6%)	349 (41.0%)	169 (41.3%)	28 (36.4%)	
**Setting type, n (%)**						< 0.001
Urban	53 (24.4%)	195 (48.5%)	544 (63.9%)	248 (60.6%)	42 (54.6%)	
Rural	164 (75.6%)	207 (51.5%)	307 (36.1%)	161 (39.4%)	35 (45.4%)	
**Maternal BMI, mean (SD)**						0.02
In Kg/m^2	25.3 (3.3)	25.9 (4.1)	26.4 (4.5)	26.1 (3.8)	25.8 (3.7)	
**Maternal BMI, n (%)**						0.10

For peer review only

Normal	108 (52.2%)	175 (46.1%)	343 (42.6%)	171 (43.4%)	33 (46.5%)
Overweight	84 (40.6%)	148 (38.9%)	333 (41.4%)	162 (41.1%)	29 (40.8%)
Obese	15 (7.2%)	57 (15.0%)	129 (16.0%)	61 (15.5%)	9 (12.7%)

SD = standard deviation; BMI = Body mass index
* Comparisons were conducted using Chi-squared tests for categorical variables, and analysis of variance for numerical ones.

For peer review only

Table 2: BMI and BMI z-score by food frequency at baseline: Mean and standard deviation (SD) during follow-up

	Mean and standard deviation of BMI and BMI z-score			
	**Baseline (n = 1948)**	**First follow-up (n = 1904)**	**Second follow-up (n = 1850)**	**Third follow-up (n = 1810)**
**Age**				
Mean age (SD)	4.3 (0.3)	6.9 (0.7)	10.9 (0.1)	13.9 (0.1)
**Food frequency**	**BMI (in Kg/m²): mean (SD)**			
< 4 times per day	16.4 (1.9)	16.4 (1.7)	18.5 (2.5)	21.1 (2.7)
4 times per day	16.4 (1.5)	16.8 (2.1)	19.4 (3.0)	21.7 (3.3)
5 times per day	16.5 (1.9)	17.0 (2.4)	19.9 (3.4)	21.8 (3.4)
6 times per day	16.3 (1.6)	16.9 (2.4)	19.8 (3.3)	21.6 (3.4)
7 times per day	16.5 (2.4)	16.8 (2.4)	19.4 (3.3)	21.0 (2.9)
**Food frequency**	**BMI z-score: mean (SD)**			
< 4 times per day	0.67 (1.06)	0.29 (0.92)	0.18 (0.92)	0.29 (0.81)
4 times per day	0.68 (0.92)	0.49 (1.02)	0.50 (1.02)	0.44 (0.93)
5 times per day	0.71 (1.09)	0.59 (1.08)	0.64 (1.11)	0.44 (1.01)
6 times per day	0.65 (0.98)	0.55 (1.13)	0.61 (1.08)	0.41 (0.99)
7 times per day	0.72 (1.33)	0.42 (1.14)	0.45 (1.15)	0.26 (0.90)

SD = Standard deviation; BMI = body mass index

For peer review only

Table 3: Food frequency and changes over time of body mass index: crude and adjusted linear mixed models

	Crude model Coefficient (95% CI)	Adjusted model* Coefficient (95% CI)
**Food frequency**	**Change in BMI (Kg/m^2)**	
< 4 times per day	**1.18 (0.73; 1.64)**	**0.39 (0.17; 0.62)**
4 times per day	**0.71 (0.43; 0.98)**	**0.16 (0.02; 0.30)**
5 times per day	1 (Reference)	1 (Reference)
6 times per day	**-0.54 (-0.68; -0.40)**	-0.03 (-0.15; 0.09)
7 times per day	**-0.73 (-1.26; -0.21)**	0.08 (-0.33; 0.50)
**Food frequency**	**Change in BMI z-score**	
< 4 times per day	0.01 (-0.08; 0.10)	**0.07 (0.01; 0.13)**
4 times per day	0.01 (-0.03; 0.06)	**0.05 (0.01; 0.09)**
5 times per day	1 (Reference)	1 (Reference)
6 times per day	0.01 (-0.03; 0.06)	-0.01 (-0.05; 0.04)
7 times per day	0.06 (-0.10; 0.21)	0.04 (-0.13; 0.20)

95% CI: 95% confidence intervals; BMI = body mass index
* Adjusted for sex, age, maternal education, paternal education, wealth index, setting type, and maternal BMI (in categories). Estimates in bold are statistical significant.

For peer review only

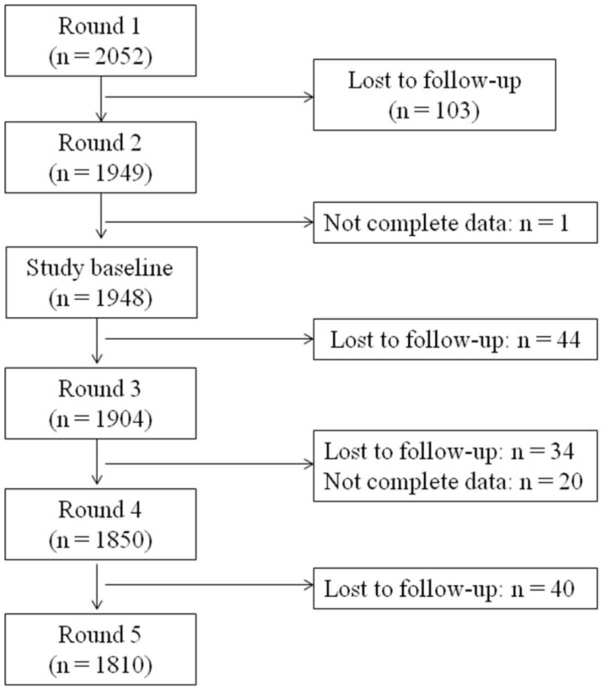

Figure 1: Flowchart of participants

150x112mm (300 x 300 DPI)

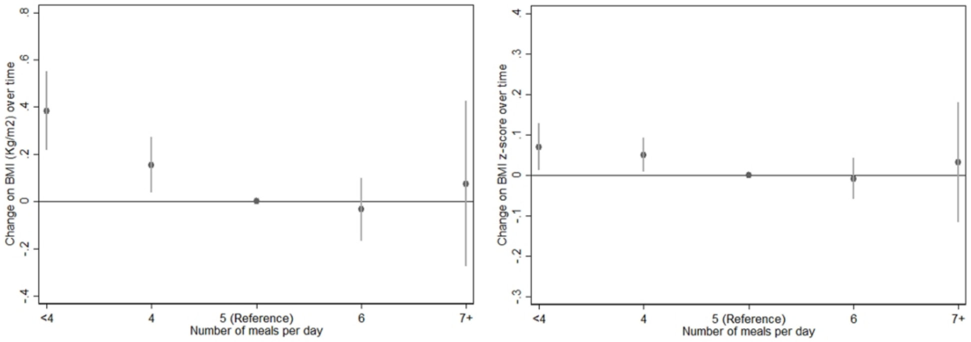

Figure 2: Changes of BMI and BMI-for-age z-score over time according to meal frequency

250x92mm (300 x 300 DPI)

STROBE 2007 (v4) Statement—Checklist of items that should be included in reports of *cohort studies*

Section/Topic	Item #	Recommendation	Reported on page #
**Title and abstract**	1	(*a*) Indicate the study's design with a commonly used term in the title or the abstract	1
		(*b*) Provide in the abstract an informative and balanced summary of what was done and what was found	3
**Introduction**			
Background/rationale	2	Explain the scientific background and rationale for the investigation being reported	6
Objectives	3	State specific objectives, including any prespecified hypotheses	6
**Methods**			
Study design	4	Present key elements of study design early in the paper	7
Setting	5	Describe the setting, locations, and relevant dates, including periods of recruitment, exposure, follow-up, and data collection	7
Participants	6	(*a*) Give the eligibility criteria, and the sources and methods of selection of participants. Describe methods of follow-up	7
		(*b*) For matched studies, give matching criteria and number of exposed and unexposed	
Variables	7	Clearly define all outcomes, exposures, predictors, potential confounders, and effect modifiers. Give diagnostic criteria, if applicable	8
Data sources/ measurement	8*	For each variable of interest, give sources of data and details of methods of assessment (measurement). Describe comparability of assessment methods if there is more than one group	8
Bias	9	Describe any efforts to address potential sources of bias	9
Study size	10	Explain how the study size was arrived at	9
Quantitative variables	11	Explain how quantitative variables were handled in the analyses. If applicable, describe which groupings were chosen and why	9
Statistical methods	12	(*a*) Describe all statistical methods, including those used to control for confounding	9
		(*b*) Describe any methods used to examine subgroups and interactions	9
		(*c*) Explain how missing data were addressed	NA
		(*d*) If applicable, explain how loss to follow-up was addressed	NA
		(*e*) Describe any sensitivity analyses	NA
**Results**			

For peer review only

Participants	13*	(a) Report numbers of individuals at each stage of study—eg numbers potentially eligible, examined for eligibility, confirmed eligible, included in the study, completing follow-up, and analysed	10
		(b) Give reasons for non-participation at each stage	10
		(c) Consider use of a flow diagram	Figure 1
Descriptive data	14*	(a) Give characteristics of study participants (eg demographic, clinical, social) and information on exposures and potential confounders	10
		(b) Indicate number of participants with missing data for each variable of interest	Figure 1
		(c) Summarise follow-up time (eg, average and total amount)	10
Outcome data	15*	Report numbers of outcome events or summary measures over time	10
Main results	16	(*a*) Give unadjusted estimates and, if applicable, confounder-adjusted estimates and their precision (eg, 95% confidence interval). Make clear which confounders were adjusted for and why they were included	10-11
		(*b*) Report category boundaries when continuous variables were categorized	10-11
		(*c*) If relevant, consider translating estimates of relative risk into absolute risk for a meaningful time period	10-11
Other analyses	17	Report other analyses done—eg analyses of subgroups and interactions, and sensitivity analyses	10-11
**Discussion**			
Key results	18	Summarise key results with reference to study objectives	11
**Limitations**			
Interpretation	20	Give a cautious overall interpretation of results considering objectives, limitations, multiplicity of analyses, results from similar studies, and other relevant evidence	11-12
Generalisability	21	Discuss the generalisability (external validity) of the study results	11-12
**Other information**			
Funding	22	Give the source of funding and the role of the funders for the present study and, if applicable, for the original study on which the present article is based	13

*Give information separately for cases and controls in case-control studies and, if applicable, for exposed and unexposed groups in cohort and cross-sectional studies.

Note: An Explanation and Elaboration article discusses each checklist item and gives methodological background and published examples of transparent reporting. The STROBE checklist is best used in conjunction with this article (freely available on the Web sites of PLoS Medicine at http://www.plosmedicine.org/, Annals of Internal Medicine at http://www.annals.org/, and Epidemiology at http://www.epidem.com/). Information on the STROBE Initiative is available at www.strobe-statement.org.

For peer review only
